# The Secretome of a Cachexia-Inducing Lung Tumor Impairs Mitochondrial Function and Skeletal Muscle Differentiation

**DOI:** 10.3390/cancers18132130

**Published:** 2026-06-30

**Authors:** Nikki Wanders, Marvin Martens, Marco Kelders, Sven Manse, Sandra van Krimpen, Claire Groenen, Chrysi Kapsali, Paula Bilbao Fraile, Konstantina Bermperi, Niels Boumans, Youssra Ahidar, Ludwig Dubois, Hubert Smeets, Wouter van de Worp, Ramon Langen

**Affiliations:** 1 Department of Translational Genomics, Mental Health and Neurosciences Research Institute (MHeNS), Maastricht University, Universiteitssingel 40, 6229 ER Maastricht, The Netherlands; n.wanders@maastrichtuniversity.nl (N.W.);; 2 Department of Translational Genomics, Research Institute of Nutrition and Translational Research in Metabolism (NUTRIM), Maastricht University, Universiteitssingel 40, 6229 ER Maastricht, The Netherlands; 3 Department of Respiratory Medicine, Research Institute of Nutrition and Translational Research in Metabolism (NUTRIM), Maastricht University, Universiteitssingel 50, 6229 ER Maastricht, The Netherlands; 4 The M-Lab, Department of Precision Medicine, Research Institute for Oncology and Reproduction (GROW), Maastricht University, Universiteitssingel 40, 6229 ER Maastricht, The Netherlands; 5 Division of Cancer Sciences, School of Medical Sciences, Faculty of Biology, Medicine and Health, The University of Manchester, Manchester M13 9PL, UK

**Keywords:** lung adenocarcinoma, C2C12, cachexia, muscle wasting

## Abstract

**Background:** Cancer-associated cachexia (CAC) can affect up to 80% of patients with late-stage cancer and is characterized by depletion of skeletal muscle mass with or without loss of fat tissue. No effective treatments are currently available, and reversing CAC requires understanding the intracellular processes of muscle atrophy and its cancer-related extracellular triggers. In this study, we aimed to disentangle tumor- and host-driven mechanisms in CAC muscle wasting. **Methods:** In skeletal muscle tissue obtained from control non-tumor-bearing mice and cachectic mice resulting from orthotopically implanted 344P lung adenocarcinoma cells, transcriptomic analyses were performed to identify muscle wasting-associated processes. To explore whether these reflected direct tumor-induced effects, 344P tumor-conditioned medium (tCM) was applied to in vitro cultured C2C12 skeletal muscle cells to investigate the impact on muscle proteolysis, myogenesis and mitochondrial function. **Results:** RNAseq data revealed increased proteolysis along with decreased myogenesis-related processes, and prominent downregulation of genes encoding mitochondrial OXPHOS complexes, in cachectic mouse muscle. Exposure of cultured skeletal muscle cells to tCM reduced mitochondrial respiration and induced changes in mitochondrial mass and mitochondrial DNA copy number. tCM did not induce myotube atrophy, or activation of proteolysis-related signaling, in fully differentiated myotubes. In contrast, tCM reversibly inhibited myoblast–myotube fusion, and reduced myogenic and muscle-specific gene expression in differentiating myoblasts. Application of CCCP to simulate muscle mitochondrial dysfunction reproduced the myogenesis-impairing phenotype caused by tCM. **Conclusions:** Our results show that factors present in the cachexia-inducing lung tumor secretome directly impair myogenesis and muscle mitochondrial function, whereas activation of muscle catabolic processes requires host-dependent mechanisms.

## Simple Summary

Cancer-associated cachexia (CAC) is a syndrome of involuntary body weight loss. Skeletal muscle tissue depletion during CAC strongly impacts the prognosis and treatment response of patients with lung cancer. We examined muscle tissue from lung tumor-bearing mice to determine which muscle-maintenance-related pathways are dysregulated in CAC. We found that pathways linked to muscle protein breakdown are increased, while processes involved in muscle regeneration and mitochondrial metabolism are decreased. In contrast, in muscle cell cultures exposed to factors secreted by the same lung tumor cells that induce CAC in mice, only muscle regeneration and mitochondrial metabolism were impaired, while muscle protein breakdown processes remained unchanged. Our results show that muscle-maintenance-related pathways deregulated in CAC can be distinguished in tumor-induced host-dependent and -independent processes. These findings provide new mechanistic insights that will guide development of therapies to prevent or reverse muscle depletion in CAC.

## 1. Introduction

Cachexia is defined as an ongoing loss of skeletal muscle mass, with or without loss of adipose tissue, due to a disease that cannot be reversed by conventional nutrition and/or exercise [1,2]. It is a multifactorial syndrome that eventually leads to a progressive decline in muscle mass and function. This decline is often neglected and is poorly manageable [2,3]. It is estimated that the prevalence of cachexia is 28–57% in cancer patients. Additionally, cancer-associated cachexia (CAC) affects up to 80% of patients diagnosed with late-stage cancer, with mortality reaching up to 22% [4,5]. Loss of muscle and adipose tissues poses a direct threat to the quality of life and treatment success of anticancer therapies, and consequently mortality rates [6]. Effective treatments to reverse cachexia are currently not available because its underlying mechanisms are not yet fully understood [4]. Current treatments mainly focus on lowering the risk of infection and modulation of nutritional intake (i.e., dietary counseling and specific nutritional supplements, and pharmacological stimulation of appetite), however these treatments have limited success due to persistent alterations in metabolism [6,7,8,9].

The mechanisms of CAC-associated muscle wasting can be divided in extracellular- and intracellular processes underlying muscle atrophy. Extracellular regulation of muscle atrophy can be caused by circulating inflammatory factors such as proinflammatory cytokines including TNF, IL-6, and leukemia inhibitory factor (LIF) [10,11]. To which extent these cytokines contribute to muscle atrophy in all cases of CAC is under debate. This has been attributed to changes in these circulating cytokines, which are not consistently elevated in every patient, possibly due to rapid clearing within the body or differences in other disease-(treatment)-related triggers [6]. Moreover, whether their increased plasma levels are caused by elevated secretion by the tumor, or are the consequence of a host‘s response to the tumor (e.g., immune cell activation) resulting in systemically elevated cytokine concentrations, is an important question that has still not been resolved. TNFα and IL-6 induce skeletal muscle atrophy following receptor binding and activation of the NF-ĸB and JAK/STAT signaling pathways, respectively, which in turn impact the intracellular processes that determine muscle mass [12]. Many preclinical studies suggest that muscle catabolism is involved in CAC-associated wasting [13,14]. RNA-seq data of cachectic mouse muscle showed upregulation of mediators of muscle breakdown, including E3-Ub-ligases (e.g., *Fbxo32*/*Atrogin1*, *Trim63*/*Murf1*); increased expression of genes blocking mTOR signaling (*Ddit4*); and changes in mediators of the autophagy–lysosomal degradation pathway [15,16,17,18].

Besides these alterations in protein turnover, impairment of nuclear turnover in myofibers has been implicated as an intracellular mechanism of muscle wasting in CAC [6]. Myonuclear turnover is the loss and addition (accretion) of myonuclei to myofibers, the latter through postnatal myogenesis [19], although this process is still not fully understood. Impaired myogenesis has been implicated in CAC based on studies showing downregulation of transcription factor *MyoD* [20,21] and alterations in expression levels of other key myogenic regulators like *myogenin and Pax7* [10,22]. Moreover, muscle fibrosis [23] and presence of small, necrotic fibers has been described in CAC, accompanied by depleting muscle progenitor cell populations [24], which are typically features of failing regeneration associated with muscular dystrophies. Accordingly, impairments in myogenesis resulting in long-term ineffective regeneration have been postulated to contribute to muscle wasting in CAC.

Alterations in mitochondrial function have been implicated in CAC [25]. Mitochondrial function is essential for the energy provision required for muscle contraction [26] and for cellular processes, like muscle protein synthesis [27,28] and myogenesis, that are involved in muscle mass maintenance [29,30,31,32]. Accordingly, impaired mitochondrial function may be involved in muscle weakness and atrophy, and mitochondrial-dysfunction-induced oxidative stress has been linked to the activation of catabolic pathways in muscle [33]. However, whether mitochondrial dysfunction per se is sufficient to induce muscle atrophy remains to be determined, since some (e.g., *OPA1* or *PolG* mutation [34,35]), but not all (e.g., *m.3243A>G* [36]) mitochondrial myopathies are accompanied by muscle mass depletion. Alternatively, muscle wasting induced by mitochondrial dysfunction may require a disease-specific context, as has been shown for sustained muscle loss following cardiac surgery [37]. In CAC it remains to be determined whether tumor- or host-derived mechanisms contribute to mitochondrial dysfunction, and which cellular processes involved in muscle mass maintenance they affect.

In previous work, we showed faithful reproduction of key features of human lung CAC following orthotopic implantation of lung adenocarcinoma 344P cells in mice, referred to as the orthotopic lung cancer cachexia (OLCC) model [38]. In that study, first indications of mitochondrial dysregulation were observed through comparative transcriptomic analysis, along with increased proteolysis and inflammatory signaling in hind limb muscles of OLCC mice. The objective of the current study was to distinguish tumor- and host-driven mechanisms of muscle wasting in CAC. To this end, we investigated which processes involved in muscle mass maintenance were affected in muscles of OLCC mice, and subsequently addressed the direct contribution of tumor-derived factors by incubating in vitro differentiating C2C12 myoblasts or C2C12 myotubes with 344P tumor cell conditioned media to evaluate the impact on mitochondrial function, muscle proteolysis signaling and myogenesis.

## 2. Materials and Methods

### 2.1. Animals

The orthotopic lung cancer cachexia (OLCC) model was induced as described previously [38]. In short, immune-competent 129S2/Sv male mice received 2 × 10^4^ cells/mL of 344P adenocarcinoma cells in 10 μL 100% Matrigel (Corning, New York, NY, USA) in the upper left lung. Sham controls (*n* = 8) received Matrigel injections only. CAC (*n* = 8) was defined as ≥5% body weight loss. Gastrocnemius (GN) muscles were isolated and snap-frozen for RNA isolation after reaching the cachexia-related endpoint. RNA was extracted using TRI reagent (Sigma-Aldrich, Saint Louis, MO, USA) according to the manufacturer’s protocol. This work (AVD1070020174168) was performed in accordance with institutional and national guidelines of Maastricht University, NL, and legislation following the European Directive 2010/63/EU.

### 2.2. RNA Sequencing

mRNA isolation was performed as described previously [38]. mRNA was isolated from 1 μg total RNA using the NEXTFLEX Poly (A) Beads 2.0 kit (PerkinElmer, Waltham, MA, USA), NEXTFLEX Rapid Directional RNA-Seq kit 2.0 and NEXTFLEX RNA-seq 2.0 Unique Dual Index Barcodes. mRNA content from CAC (*n* = 4) and sham (*n* = 4) mouse muscles were sequenced with a NovaSec 6000 system (Illumina, San Diego, CA, USA) using an S2 flow cell (Illumina).

### 2.3. Bioinformatics Analysis

Differential expression analysis was performed using DESeq2 [39]. Genes with FDR < 0.01 and |log2FC| ≥ 0.5 were considered differentially expressed, yielding 2974 DEGs from 11,599 expressed genes.

Gene Ontology analysis: Gene-to-GO annotations were retrieved from the QuickGO REST API (EBI; https://www.ebi.ac.uk/QuickGO/ (accessed on 13 April 2026)) for Mus musculus (taxonId = 10,090, Biological Process aspect). The GO ontology structure (go-basic.obo, December 2025 release, 48,201 terms) was parsed using the Pronto library to identify direct children using “is_a” and “part_of” relationship types. For exploring myogenesis-related processes, parent terms GO:0042692 (muscle cell differentiation) and GO:0007519 (skeletal muscle tissue development) were used, and for proteolysis the parent term GO:0006508 (proteolysis) was used, along with their direct child terms. To complement the GO-based analysis, a literature-curated myogenesis gene set was compiled comprising 56 genes across seven functional categories: myogenic regulatory factors, myosin heavy chains, myosin light chains, actin cytoskeleton and troponin complex components; sarcomere and structural proteins; satellite cell markers; and growth factors and signaling molecules. A full list of selected gene sets can be found in Supplementary Table S3.

GSEA/unbiased analysis: Over-representation analysis of all 2974 DEGs (Ensembl gene IDs, 2660 mapped to annotated genes) was performed with g:Profiler (g:GOSt, Mus musculus), restricted to GO Biological Process, with Benjamini–Hochberg FDR < 0.05. Pre-ranked GSEA was performed with gseapy (v1.1.4) on all expressed genes, ranked by the signed DESeq2 Wald statistic, against GO Biological Process gene sets (Enrichr GO_Biological_Process_2023, gene sets of 15–500 genes, 1000 permutations).

Upstream regulator analysis: Transcription factor–target interactions were obtained from TFLink (v1.0), restricted to literature-curated (small-scale evidence, e.g., EMSA, DNase-I footprinting, SELEX, promoter-deletion reporter assays) interactions. For each transcription factor with at least 10 curated targets among expressed genes, enrichment of its targets within the proteolysis DEGs (GO:0006508, *n* = 163) and, separately, the myogenesis DEGs (GO:0042692 and GO:0007519, *n* = 36) was assessed by one-sided Fisher’s exact test against the background of all 11,599 expressed genes. The ten transcription factors most strongly enriched per process (lowest enrichment *p*-value) were reported. The directional specificity of the proteolysis signal was confirmed by repeating the test on the up- and downregulated proteolysis DEGs separately. The results were visualized as a directed TF-target network combining both proteolysis and myogenesis.

Mitochondrial gene annotation: Mitochondrial genes were annotated using MitoCarta 3.0 [40], a comprehensive inventory of mammalian mitochondrial proteins containing 1140 mouse genes. The ‘MitoCarta3.0_MitoPathways’ column contains hierarchical pathway information with pipe-delimited (‘|’) multiple pathways and greater-than symbols (‘>’) separating hierarchical levels. MitoCarta 3.0 assigns genes to hierarchical pathways, meaning that a single gene may be annotated to multiple sub-pathways within the same parent category. Gene counts per top-level category represent unique genes. In the hierarchical pathway analysis, genes are counted once per pathway assignment to reflect representation across individual sub-pathways.

Statistical testing: Pathway enrichment and directional bias were assessed using Fisher’s exact test with Benjamini–Hochberg FDR correction. Enrichment tests (one-tailed) compared DEG rates in pathways to genome-wide rates (2974/11,599 = 25.6%). Directional bias tests (two-tailed) compared up/down ratios within each pathway to the global distribution (55.5% upregulated/44.5% downregulated). FDR correction was applied separately by hierarchical level and test type to account for differences in pathway sizes across levels. Statistical analyses were performed using Python 3.x with scipy (Fisher’s exact test) and statsmodels (FDR correction). Custom scripts used for downstream analysis and figure generation are publicly available on Zenodo (v1.1.0) [41].

### 2.4. Cell Culture

All cells were maintained in a humidified incubator at 37C with 5% CO_2_. C2C12 mouse myoblasts (#CRL-1772, ATCC, Manassas, VA, USA) were kept in growth medium (GM) consisting of Dulbecco’s modified Eagle’s medium low glucose (DMEM 1 g/L glucose) (#22320-022, Gibco, Grand Island, NY, USA), supplemented with 9% heat-inactivated fetal bovine serum (hiFBS, ThermoFisher, Waltham, MO, USA) and 0.5% 100× Penicillin/Streptomycin (p/s, ThermoFisher). Differentiation was induced by exposing >90% confluent C2C12 on 2% Matrigel-coated tissue culture plates to differentiation medium (DM) consisting of DMEM high glucose (4.5 g/L glucose, #41966-029, Gibco), supplemented with 0.5% hiFBS and 0.5% p/s for 5 days. Postnatal myonuclear accretion was assessed via luciferase activity in Cre-recombinase-expressing C2C12 and LV-floxed-Luc-expressing C2C12, as described previously [42]. In short: Cre-IRES-PuroR (#30205, Addgene, Watertown, MO, USA) was incorporated into C2C12 myoblasts via lentiviral transduction. LV-floxed-Luc (#60622, Addgene) was incorporated through transfection via Lipofectamine 2000 (Invitrogen, Carlsbad, CA, USA).

Murine 344P and 393P lung adenocarcinoma cells (p53^R172HΔg/+^ K-ras^LA1/+^) were provided courtesy of Prof. Dr. JM Kurie, University of Texas, MD Anderson Cancer Center. Cells were maintained in RPMI 1640 medium 2 g/L glucose (#11875093, Gibco), and supplemented with 9% FBS (ThermoFisher) and 0.5% p/s. Murine C10 lung epithelial cells [43] were kept in CRML 1066 medium 1 g/L glucose (#11530037, Gibco), and supplemented with 10% hiFBS, 1% 100× p/s and 1% L-Glutamine (Invitrogen). Conditioned medium (CM) consisted of C2C12 DM high glucose, and was incubated with 2700 cells/cm^2^ 344P or C10 for 48 h to obtain tumor tCM and control cCM, respectively. The medium was stored at −20 °C after centrifugation (400× *g* for 5 min). C2C12 was cultured in undiluted CM for most experiments. To test the impact of glucose depletion (~30% in tCM, and 10% in cCM), glucose was supplemented to the standard concentration of 4.5 g/L in DMEM. Experiments involving the mitochondria uncoupler carbonyl cyanide m-chlorophenyl hydrazone (CCCP) consisted of CCCP dissolved in DMSO (vehicle), with a final working solution of 3 µM in DM.

### 2.5. Postnatal Myonuclear Accretion Detection via Luciferase Activity Measurement

Cell lysates in reporter lysis buffer (Promega, Madison, WI, USA) were stored at −20 °C and freeze-thawed before measuring. Luciferase assay reagent (LAR; 1.07 mM MgCO_3_, 2.67 mM MgSO_4_, 20.0 mM Tricin, 0.10 mM EDTA, 33.3 mM DTT, 530 μM ATP, 270 μM Coenzyme A, 470 μM Luciferin) was added to the cell lysate. Relative light unit (RLU) was measured (10 s. integration time, 333 μL/s. injection rate, 0.4 s. interval) via Promega GloMax and corrected by protein content via BCA protein assay (Pierce Biotechnology, Waltham, MO, USA).

### 2.6. Quantitative PCR

RNA from cells was isolated via TRI-reagent (Sigma-Aldrich) according to the manufacturer’s protocol. 1-Bromo-3-chloropropane (Sigma-Aldrich) was used instead of Chloroform. Glycogen (ThermoFisher) was added as a co-precipitant. A cDNA synthesis kit (GC-Biotech, Waddinxveen, The Netherlands) was used to obtain cDNA from 400 ng single-stranded RNA via reverse transcription according to the manufacturer’s protocol. DNA for mitochondrial copy number quantification was isolated via a Promega DNA isolation kit (Promega). Realtime quantitative PCR was performed using the LightCycler 480 (Roche Life Sciences, Basel, Switzerland) system for 10 min at 95 °C, followed by 45 cycles of 10 s at 95 °C and 20 s at 60 °C, followed by a melting curve determination cycle. Relative gene expression was calculated using LinRegPCR version 2021.2 (Amsterdam UMC, Amsterdam, The Netherlands). The primers are listed in Supplementary Table S1.

### 2.7. Protein Isolation and Western Blot

The methods employed for this analysis are described in [38]. In short, muscle powder was homogenized in whole-cell lysate buffer (20 mM Tris, 120 mM NaCl, 1% Nonidet, 1 mM DTT, PhosSTOP and complete mini protease inhibitor cocktail). Samples were taken up in Laemmli buffer (1:4) for 5 min at 95 °C. Antibodies consisted of Total OXPHOS Rodent WB Antibody (#AB110413, Abcam, Cambridge, UK, 1:1000 in 0.05% Tween-20) and secondary antibodies (anti-mouse IgG peroxidase [1:10,000]). The samples were imaged with an Amersham Imager 600 (GE Life Sciences, Princeton, NJ, USA), and quantified using ImageQuant TL 1D software version 8.1 (GE Life Sciences).

### 2.8. Seahorse Cell Mito Stress Test

C2C12 myoblasts were plated at an optimized density of 5000 cells/well in a XFe24 plate in a Seahorse XFe24 analyzer (Agilent Technologies, Santa Clara, CA, USA). After reaching >90% confluency, the medium was switched to DM for 5 days to obtain myotubes. Subsequently, culture medium was replaced with DM, tCM or cCM for an additional 3 days. On the day of measurement, plates were equilibrated for 1 h with Seahorse XF DMEM Assay medium (Agilent) supplemented with 10 mM glucose, 2 mM glutamine and 1 mM pyruvate. Next, compounds of the Mito Stress Test were successively injected and oxygen consumption rate (OCR) was assessed. Details of the composition of working solutions are provided in Supplementary Table S2. Values were corrected by protein content via BCA protein assay (Pierce Biotechnology) according to the manufacturer’s protocol.

### 2.9. Energy Production

Cellular adenosine triphosphate (ATP) content was measured via a CellTiterGlo Luminescent Cell Viability assay (Promega) according to the manufacturer’s protocol. Measurements were performed in a microplate luminometer (Glomax, Promega). ATP was corrected for DNA content from the same well by Quant-iT PicoGreen dsDNA Assay kit (Invitrogen) according to the manufacturer’s protocol. Measurements were performed using a microplate fluorimeter (Isogen Life Sciences, De Meern, The Netherlands).

### 2.10. Enzymatic Assays

Citrate synthase (CS) activity was determined as a measurement of mitochondrial mass [44]. Cell lysates of treated myotubes were collected in 0.5% Triton X-100. The CS reagent consisted of Tris base (12 mg/mL), 5,5-ditio-bis-(2-nitrobenzoic acid) (DTNB, 0.04 mg/mL), and Acetyl CoA (0.04 mg/mL), dissolved in H_2_O (pH 8). The starting reagent consisted of oxaloacetic acid (3.3 mg/mL in H_2_O). Activity was measured on a spectrophotometer at 412 nm at 37 °C.

### 2.11. Statistics

Statistical analysis for the experimental procedures was performed via GraphPad Prism 8 software (Dotmatics, Boston, MA, USA). Experiments were replicated a minimum of three times, with representative results shown here or specified as a nested test. Values were tested for normality (Shapiro–Wilk) and homoscedasticity beforehand. Comparisons of two groups were statistically tested with a two-tailed unpaired T-test for normally distributed data or a Mann–Whitney U test for non-parametric data. Data with more than two groups were statistically tested through Ordinary One-way ANOVA with Tukey’s correction when considered normally distributed. A Kruskal–Wallis test with Dunn’s correction was used for non-parametric data.

## 3. Results

### 3.1. Altered Regulation of Processes Related to Proteolysis and Myogenesis in Skeletal Muscle of Cachectic Lung Tumor-Bearing Mice

Lung orthotopic inoculation of 344P adenocarcinoma cells resulted in a cachectic phenotype as previously published for this OLCC model, including loss of body weight (Δ-15%, *p* ≤ 0.05), reduced grip strength (Δ-44%, *p* ≤ 0.05), and muscle wasting (−13% muscle weight loss, *p* = 0.057) compared to healthy sham mice (Supplementary Figure S1, [38]). mRNA content in muscles of OLCC mice was compared to that in healthy sham mice. Differential expression analysis identified 2974 DEGs (55.5% upregulated) among 11,599 expressed genes. To characterize the transcriptional remodeling before any candidate-driven focus, we first explored the genome-wide enrichment landscape (Supplementary Figure S2A, [41]). Over-representation analysis of all DEGs identified broad dysregulation spanning programmed cell death, ribosome biogenesis, nucleotide metabolism, protein modification by ubiquitin-like conjugation, NF-κB signaling, immune cell differentiation, RNA splicing and muscle cell differentiation. Gene set enrichment analysis on the ranked gene list resolved the direction of these changes: RNA processing/splicing, ribosome biogenesis and translation were enriched among upregulated genes, whereas oxidative phosphorylation and mitochondrial respiration showed the strongest enrichment of all among downregulated genes (Supplementary Figure S2B). Because of these results and the established role of these processes in muscle wasting, we next focused on proteolysis, myogenesis and mitochondrial function in detail.

**Figure 1:**
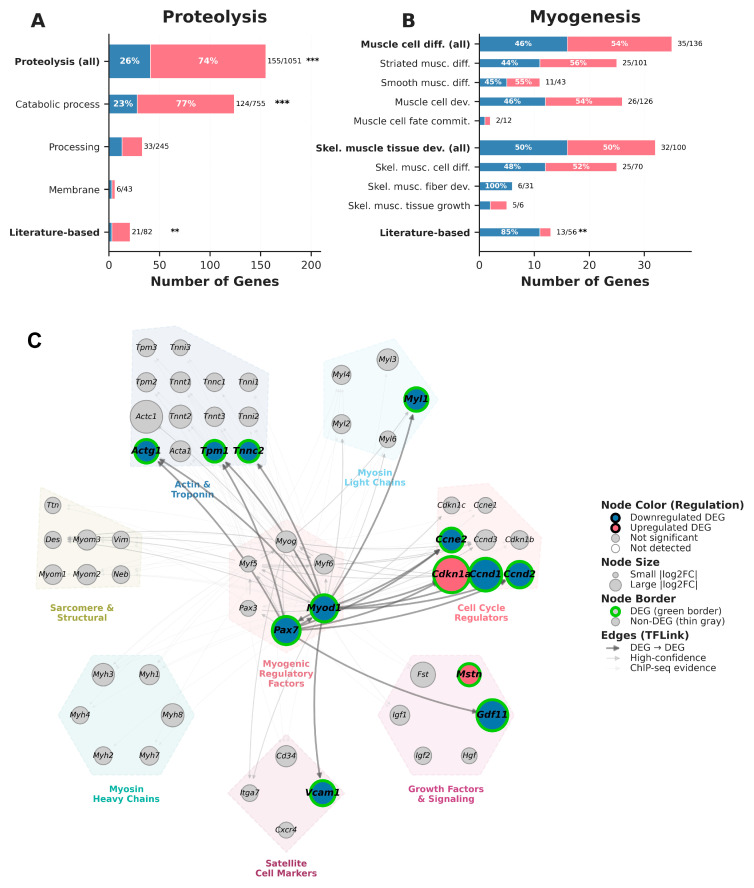
Differential expression analysis of genes involved in processes related to proteolysis (**A**) and myogenesis (**B**) in gastrocnemius muscles of OLCC mice compared to sham control mice. Upregulated genes are depicted in red; downregulated DEGs are depicted in blue. Directional bias was assessed by Fisher’s exact test (two-tailed) with FDR correction. A literature-based approach for atrophy-related genes yielded 82 genes. A literature-based search for genes related to myogenic processes yielded 56 genes, shown in a regulatory network with interconnecting nodes. Node size indicates the number of log 2-fold changes; green node border indicates DEGs. Edges represent TF–target interactions from TFLink, supported by small-scale evidence or by high-throughput ChIP-seq (**C**). Edges with at least one small-scale record are drawn thicker (higher confidence). Edges supported only by ChIP-seq are drawn thinner. ** FDR ≤ 0.005, *** FDR ≤ 0.001.

Within the parent term Proteolysis (GO:0006508), 155 DEGs from 1051 annotated genes were identified. Subcategory analysis revealed protein catabolism (GO:0051603) as the dominant process, with 124 DEGs (80% of all proteolysis), 77% of which were upregulated in GN muscles (Figure 1A). The literature-based atrogene signature gene set showed that atrogenes *Fbxo32/Atrogin-1* and *Trim63/MuRF1* were among the most strongly upregulated DEGs in the entire dataset (Supplementary Table S3).

Follow-up analysis of myogenesis-related processes included two parent GO-terms, namely (1) Muscle cell differentiation (GO:0042692, 35 DEGs) and (2) Skeletal muscle tissue development (GO:0007519, 32 DEGs). A balanced pattern of up- and downregulation was observed in both parent GO-terms, with only skeletal muscle fiber development being uniformly downregulated in six significant DEGs (Figure 1B). In addition, a manual literature-based approach including known myogenesis-related genes (Supplementary Table S4) resulted in 13/56 (23.2%) DEGs in our dataset, with 37/56 (66.1%) genes unchanged and 6/56 (10.7%) undetected. Nevertheless, strong coordinated downregulation of 84.6% (*n* = 11/13, FDR < 0.01) was found in key myogenesis regulators (*Myod1*, *Pax7*), cell-cycle genes (*Ccnd1/2*↓, *p21*↑), and muscle structural proteins (*Tnnc2*, *Tpm1*, *Myl1*, *Actg1*), with nodes connected via edges, i.e., showing interaction within the network with the key genes *Pax7* and *MyoD* (Figure 1C). Combined, this data illustrates activation of proteolysis- and suppression of myogenesis-related processes in atrophying skeletal muscle of OLCC mice.

To identify the transcriptional regulators of these processes, we tested which transcription factors preferentially target the proteolysis and myogenesis DEGs using literature-curated TFLink interactions. The top ten regulators of proteolysis DEGs were dominated by the NF-κB family (*Bcl3*, *Rel*, *Rela*), STAT factors (*Stat3*, *Stat1*), *Trp53*, *Egr1*, *Foxo1*, *Cebpb* and *Ets1* (Supplementary Figure S3, [41]). This enrichment was confined to the upregulated proteolysis genes, as no transcription factors were enriched among the downregulated subset.

The top ten regulators of myogenesis DEGs combined myogenic master factors (*Pitx2*, *Pax3* and the *MyoD* coactivator *Ep300*, plus *Usf1/Usf2* and *Myb*) with inflammatory/stress regulators (*Stat3*, *Trp53*, *Ets1*), which also rank among the top ten regulators of proteolysis, indicating that a common host-driven signaling axis marks both processes (Supplementary Figure S3). These three shared regulators are shown to bridge the two processes in a combined regulatory network, coordinating upregulation of proteolysis- and downregulation of myogenesis-related processes.

### 3.2. Inhibition of Myogenesis by Lung Tumor-Derived Factors

**Figure 2:**
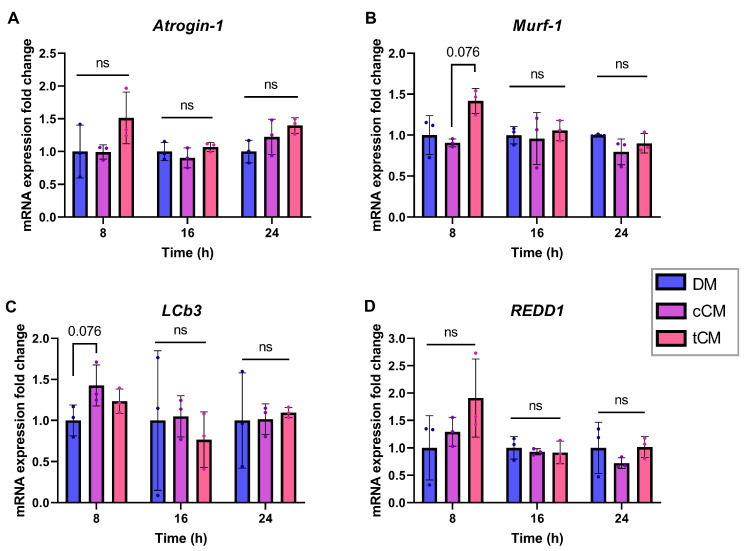
mRNA expression levels of regulatory molecules of protein turnover in C2C12 myotubes remain unaffected in response to tumor secretome. C2C12 myoblasts were differentiated for 5 days with reference (DM), cCM or tCM for 8, 16 and 24 h. mRNA expression levels of atrophy-related markers *Atrogin-1* and *Murf-1* (**A**,**B**), autophagy marker *LC3b* (**C**) and protein synthesis regulator *REDD1* (**D**). Kruskal–Wallis test with Dunn’s correction. ns = non-significant.

The observations in the skeletal muscle transcriptome prompted us to investigate whether these atrophy-associated changes may reflect the actions of factors secreted by the tumor into the circulation. To test whether tumor-secreted factors directly induce proteolysis or impair myogenesis, C2C12 myotubes or myoblasts were exposed to tCM containing lung tumor secretome obtained from the in vivo cachexia-inducing 344P cell line. No significant differences were observed in the expression of key regulatory enzymes of muscle proteolysis, i.e., *Atrogin-1* or *Murf-1*, when C2C12 myotubes were exposed to (tumor) conditioned medium (CM) for either 8, 16 or 24 h (Figure 2). Additionally, no differences were apparent in autophagy (*LC3b*) and protein synthesis regulatory (*REDD1*) processes between cCM and tCM. Accordingly, no atrophy was observed in myotubes exposed to tCM for up to 72 h (Supplementary Figure S4), although myotube cultures were receptive to atrophy-inducing stimuli, as dexamethasone treatment evoked clear myotube atrophy and atrogene induction (Supplementary Figure S4). Here, we have shown that proteolysis processes in differentiated C2C12 myotubes remained unaffected after exposure to solely tumor-associated factors released by CAC-inducing tumor cells.

**Figure 3:**
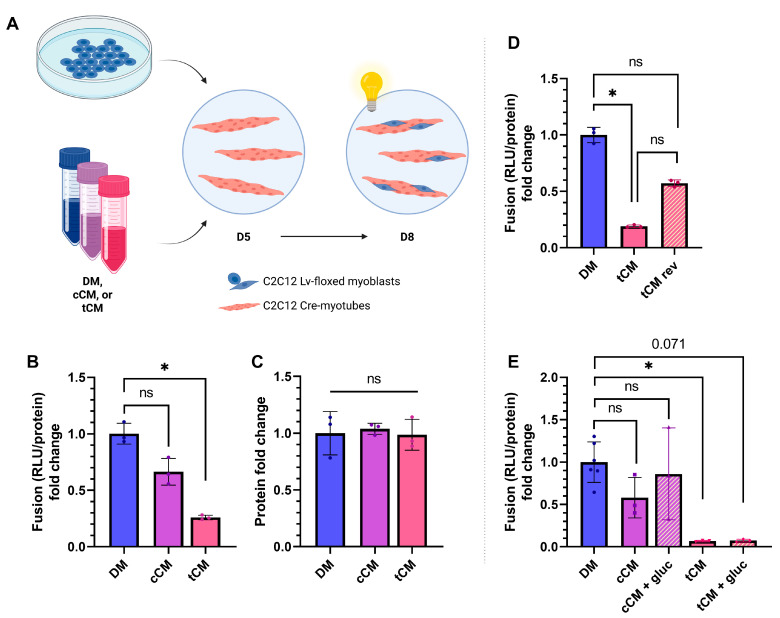
Tumor secretome decreases in fusion capacity of myoblasts with differentiated myotubes. Experimental design of post-nuclear accretion (PMA) with reporter system (**A**). PMA after 72 h exposure to reference (DM), cCM and tCM (**B**) and corresponding protein levels (**C**). Nested One-Way ANOVA with Tukey correction. PMA after exposure to DM and tCM for 24 h followed by additional recovery (rev) of 48 h in DM. Kruskal–Wallis test with Dunn’s correction. (**D**). PMA after exposure to DM, cCM and tCM with or without glucose (gluc) supplementation. Kruskal–Wallis test with Dunn’s correction. (**E**). * *p*-value ≤ 0.05. ns = non-significant.

Next, we investigated whether the suppression of myogenesis-related processes in cachectic mice represented a direct effect of tumor-secreted factors on skeletal muscle. Mimicking postnatal myonuclear accretion (PMA), as an important process of myogenesis in adult muscle, using our previously described reporter system [42] (Figure 3A), a significant decrease in myoblast-to-myotube fusion (Δ74%) was observed when cultured in the presence of tCM compared to DM (Figure 3B), with similar protein content observed between groups (Figure 3C). A similar effect was observed for tCM derived from a second cachexia-inducing lung tumor cell line (393P, Supplementary Figure S5), suggesting inhibition of PMA is a preserved mechanism in the presence of cachexia-associated muscle wasting factors. Removal of lung tumor factors by replacing CM with DM partly reversed this effect, indicating the inhibition was unlikely to result from cell death (Figure 3D). In addition, glucose supplementation (Figure 3E) did not reverse the tCM reduction in fusion (tCM, 0.07 ± 0.01 vs. tCM + gluc 0.07 ± 0.02, ns), ruling out nutrient depletion as the cause of tumor-induced inhibition of myonuclear accretion.

**Figure 4:**
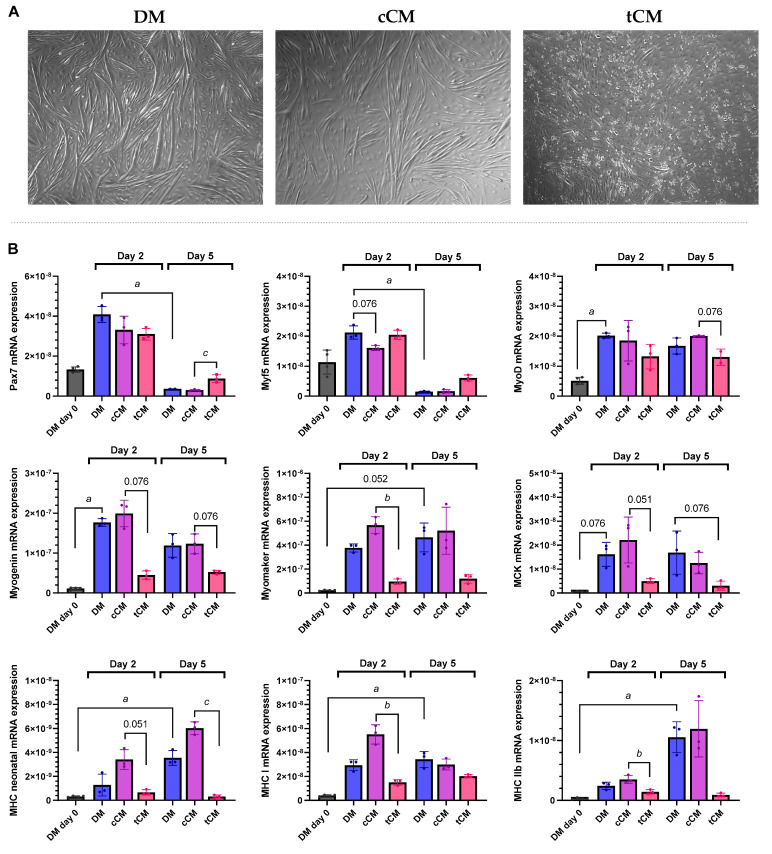
Reduced mRNA expression levels of myogenic markers and muscle-specific genes in differentiating C2C12 in response to tumor secretome. Phase contrast images of C2C12 myotubes after 5 days of differentiation with reference (DM), cCM or tCM (**A**). Expression levels of myogenic markers were measured via quantitative PCR in parental C2C12 after 0, 2 or 5 days of differentiation. Statistical relevance (*p*-value ≤ 0.05) between normal differentiation conditions of DM over time was visualized with *a*, and analysis was performed via Kruskal–Wallis test with Dunn’s correction. Statistical relevance (*p*-value ≤ 0.05) of comparisons within day 2 and 5 are visualized with *b* and *c*, respectively, and analysis was performed via Kruskal–Wallis test with Dunn’s correction (**B**).

In addition, when administered to differentiating myoblasts, tCM inhibited myotube formation (Figure 4A) and affected gene expression levels of myogenic commitment (i.e., *Pax7*) and early myogenic differentiation markers, e.g., *Myogenin* and *MyoD* (Figure 4B). *Myomaker* expression at days 2 and 5 was suppressed after tCM exposure (Figure 4B), in line with the reduction in fusion and myotube formation (Figure 4A). Furthermore, a consistent reduction in the expression patterns of muscle-specific genes, e.g., *MCK* and myosin heavy chains (MHCs) was observed in cultures exposed to tCM compared to cCM or DM (Figure 4B). Combined, these data do not provide evidence that proteolytic processes are directly induced by tumor-derived factors, but highlight compromised myocyte fusion and impaired myogenic differentiation in response to the secretome of cachexia-inducing tumors.

### 3.3. Mitochondrial Dysregulation in Skeletal Muscles of Cachectic Mice

**Figure 5:**
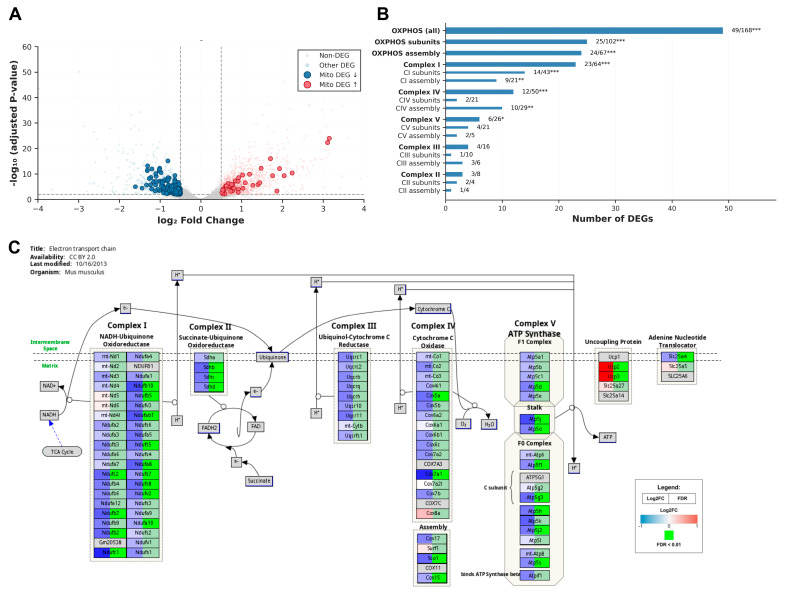
Oxidative phosphorylation pathway regulation analyzed using MitoCarta 3.0 pathway annotations. (**A**) Volcano plot of differential gene expression with mitochondrial genes (MitoCarta 3.0) highlighted. Blue indicates downregulated genes, and red indicates upregulated genes. (**B**) Hierarchical analysis of OXPHOS-related DEGs. Directional bias assessed by Fisher’s exact test (two-tailed) with Benjamini–Hochberg FDR correction. * FDR ≤ 0.05. ** FDR ≤ 0.005, *** FDR ≤ 0.001. (**C**) PathVisio visualization of DEGs mapped onto the Mus musculus electron transport chain pathway from WikiPathways (WP295); log2 fold change is indicated on the left side of gene nodes (blue: downregulated, red: upregulated), statistical significance (FDR < 0.01) is indicated in bright green on the right side.

To evaluate whether mitochondrial dysfunction contributes to muscle atrophy, the muscle transcriptome of CAC and healthy sham mice was assessed for alterations in mitochondria-related gene expression (Figure 5A). Further analysis of alterations in mitochondria-related processes (as shown by our genome-wide enrichment landscape; Supplementary Figure S2) revealed predominantly downregulated expression of mitochondrial genes (200 of 240) in OLCC muscle, with the upregulated minority enriched for fatty acid oxidation and ketogenic enzymes (e.g., *Pdk4*, *Acot2*, *Hmgcs2*, *Ucp3*). Significant downregulation was observed in pathways including translation (40 DEGs, 97.5% down, FDR < 0.001), small-molecule transport (19 DEGs, 73.7% down, FDR < 0.05) and metabolism (118 DEGs, 80.5% down, FDR < 0.05). Mitochondrial pathway analysis (full analysis provided in Supplementary Figure S6) showed significant downregulation of OXPHOS-related processes (49/168 DEGs, FDR < 0.001), which was further highlighted in a hierarchical analysis (Figure 5B), revealing suppressed expression levels of genes encoding or involved in subunit assembly of Complexes I-V and possibly suppression of the oxidative phosphorylation machinery.

**Figure 6:**
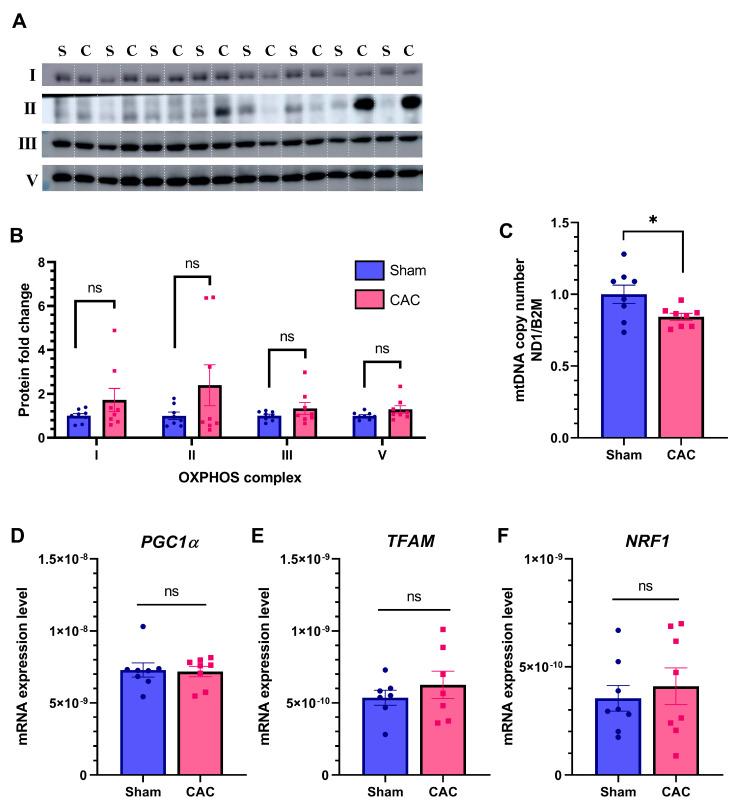
Mitochondrial regulation in gastrocnemius muscle of CAC mice (C) and sham (S). Western blot was performed as previously described to determine protein levels of OXPHOS complexes (39). Protein content was normalized by Ponceau S staining (**A**). Complex V (55 kDa), Complex III (48 kDa), Complex II (30 kDa) and Complex I (20 kDa) are visualized as fold change compared to sham mice (**B**). Mitochondrial copy number is depicted as fold change in ND1/B2M levels compared to sham (**C**). mRNA expression levels of key mitochondrial biogenesis regulators in OLCC mice (**D**–**F**). Data is represented as mean ± SEM. Mann–Whitney test. * *p*-value ≤ 0.05. ns = non-significant.

WikiPathway visualization in *Mus Musculus* (Figure 5C) was used as functional representation of DEGs, highlighting the predominant downregulation of mitochondria-related gene expression and specifically that of subunits of OXPHOS complexes in atrophied muscle of CAC mice. However, protein levels of OXPHOS complexes remained unchanged in these muscles (Figure 6A,B). The uncropped blots and molecular weight markers are shown in Supplementary Figure S7. Mitochondrial DNA content was slightly reduced in CAC mice, as shown by a decrease in copy number (*ND1/B2M)* (Figure 6C), yet key regulators for mitochondrial biogenesis (*PGC1α*, *TFAM*, *NRF1*) remained unchanged (Figure 6D–F). Nevertheless, the significant mitochondrial downregulations observed in the muscle transcriptome suggested loss of coordination of mitochondrial-related processes in muscles of 344P tumor-bearing CAC mice, which prompted us to continue to investigate any direct, causal relationships between the 344P tumor secretome, mitochondrial dysfunction and muscle mass regulation in vitro.

### 3.4. Impact of Tumor-Derived Factors on Mitochondrial Processes and Function in Muscle Cells

**Figure 7:**
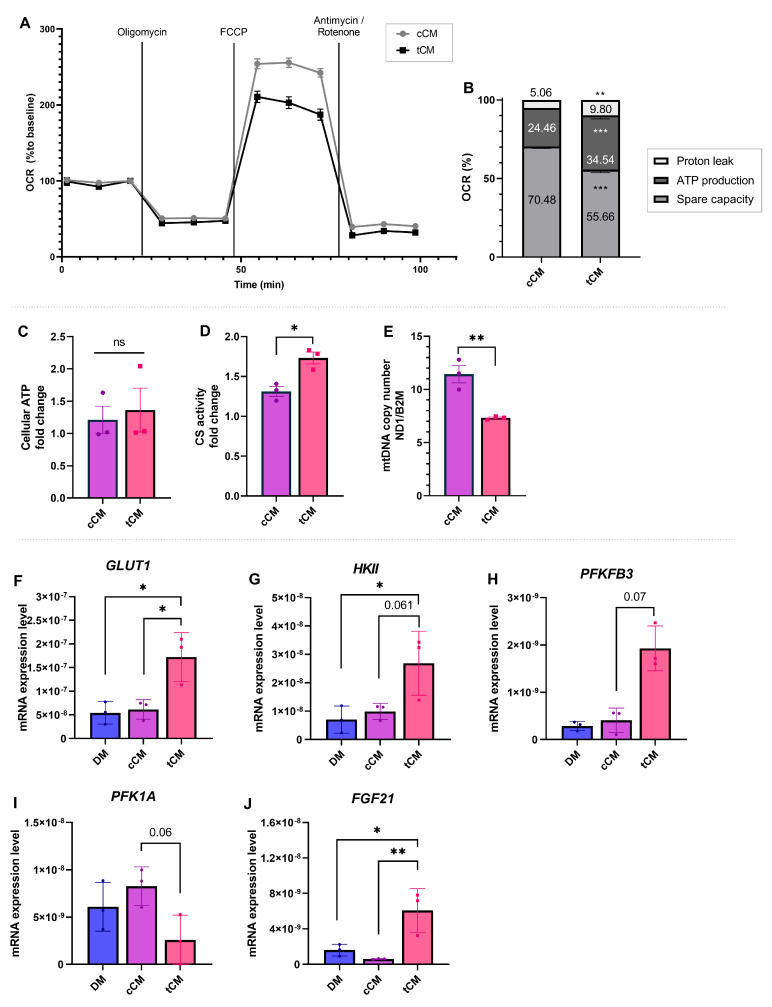
Tumor-conditioned medium induced loss of coordination of mitochondrial processes in C2C12 myotubes. C2C12 cells were differentiated for 5 days followed by 24 h of additional incubation in absence (DM) or presence of cCM or tCM. Mitochondrial respiration was assessed using Seahorse analyzer normalized to protein content, and results are presented as percentage of last basal measurement (**A**). (**B**) Stacked plot of spare respiratory capacity, ATP production and proton leak. Values (*n* = 7/group) are shown as mean ± SEM. Additionally, mitochondrial parameters including cellular ATP (**C**), oxidative capacity (**D**) and mitochondrial copy number (**E**) were assessed and are expressed as fold change compared to reference (DM). mRNA expression levels of glycolytic markers (**F**–**I**) or the mitochondrial stress marker FGF21 (**J**) were also assessed. (**B**–**D**) Unpaired T-test, Mann–Whitney; (**F**,**G**,**I**,**J**): One-way ANOVA, Tukey correction; H: Kruskal–Wallis, Dunn’s correction. * *p*-value ≤ 0.05, ** *p*-value ≤ 0.01, *** *p*-value ≤ 0.005. ns = non-significant.

To determine if tumor-derived factors impacted mitochondrial respiration, the oxygen consumption rate (OCR) of myotubes exposed to CMs was measured (Figure 7A). No morphological differences were observed in C2C12 myotubes after exposure to DM, cCM or tCM for 72 h, but total protein was increased in DM compared to both CM conditions (Supplementary Figure S8). Basal respiration levels were similar in cCM and tCM and were both set to 100% (Supplementary Figure S8). Spare respiratory capacity (SRC) in tCM (56%) was reduced compared to cCM (70%) along with increases in protein leak and ATP-related OCR (Figure 7B). Non-mitochondrial respiration (Δ0.79 pmol/min/Norm; unit, *p*-value = 0.053) and maximal respiration (Δ4.01 pmol/min/Norm; unit, *p*-value = 0.097) showed a decreasing trend in tCM, yet no differences were observed in coupling efficiency between cCM and tCM (Supplementary Figure S8). Surprisingly, cellular ATP content, which strongly relies on mitochondrial respiration in muscle, was not affected by exposure to tCM when compared to cCM (Figure 7C). CS activity, as a marker for oxidative capacity, was increased in tCM-exposed C2C12 (Figure 7D). Conversely, mitochondrial DNA copy number decreased in response to tCM by almost half (Figure 7E).

Next, gene expression levels related to glycolysis in response to tCM were examined. Increased levels of *GLUT1* and trends of elevated *HKII* and *PFKF3B* in tCM compared to cCM were seen (Figure 7F–H), along with a 3-fold reduction in *PFK1A* in tCM (Figure 7I). The metabolic switch towards glycolysis after tCM exposure suggested by this data was in line with the observed reduction in SRC. Lastly, expression of *FGF21* was induced in tCM exposed C2C12, indicative of a metabolic stress response of skeletal muscle cells following tumor secretome exposure (Figure 7J).

**Figure 8:**
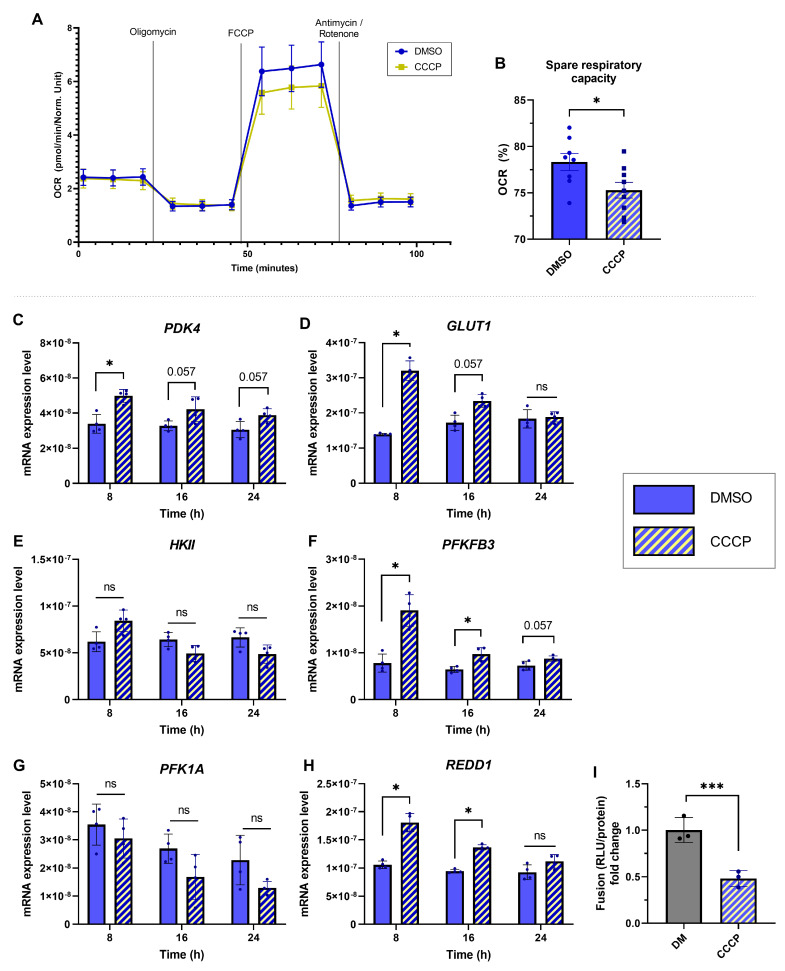
Disruption of muscle mitochondrial function impairs myogenesis. (**A**) Oxygen consumption rate (OCR) corrected for protein content. Values (*n* = 8–9/group) are shown as mean ± SEM. Spare respiratory capacity shown as a percentage of total OCR (ATP/SRC/proton leak). (**B**) Gene expression levels of glycolysis (**C**–**G**) or REDD1 protein synthesis (**H**) regulatory genes after CCCP exposure for 8, 16 and 24 h. (**I**) Myogenic fusion capacity of C2C12 myoblasts with C2C12 myotubes in presence or absence of 72 h CCCP. Values represent mean ± SD. (**B**–**H**) Mann–Whitney; (**I**) Nested T-test. * *p*-value ≤ 0.05, *** *p*-value ≤ 0.001. ns = non-significant.

The pharmacological mitochondrial uncoupler CCCP was used to emulate mitochondrial dysfunction observed in muscle cells in response to tCM. CCCP effectively disrupted mitochondrial respiration in myotubes, evidenced by reduced OCR (Figure 8A). Moreover, though it did not affect *HKII* and *PFK1A*, CCCP evoked changes in expression of *PDK4*, *GLUT1*, and *PFKFB3*, suggestive of compensatory upregulation of glycolysis compared to the vehicle control (Figure 8B–G). While CCCP did not increase muscle proteolysis-associated gene expression (Supplementary Figure S9), increased expression of *REDD1* at 8 and 16 h implied suppression of protein synthesis (Figure 8H). Importantly, CCCP strongly reduced myoblast fusion with existing myotubes (Figure 8I) consistent with the hypothesis that proper mitochondrial function is required for myonuclear accretion, and implying tCM-induced mitochondrial dysfunction as a potential mechanism by which the tumor secretome impairs myogenesis and contributes to muscle wasting in CAC.

## 4. Discussion

The objective of the current study was to distinguish tumor- and host-driven mechanisms of muscle wasting in CAC, specifically focusing on mitochondrial dysfunction, as well as alterations in proteolysis and myogenesis. Transcriptomic analysis performed in skeletal muscle tissue from control and OLCC mice identified processes indicative of increased muscle catabolism and downregulation of myogenesis and mitochondrial function in lung cancer-associated muscle wasting. To distinguish direct, tumor-induced and host-dependent effects, these processes were evaluated by differentiating C2C12 myoblasts, and C2C12 myotubes were exposed to tCM. Our results show that factors present in the cachexia-inducing lung tumor secretome directly impair myogenesis and muscle mitochondrial function, whereas activation of muscle catabolic processes is likely attributable to host-driven mechanisms.

Muscle atrophy in CAC is generally considered a consequence of a disbalance between muscle protein synthesis and degradation, with evidence predominantly suggesting increased proteolysis [6,33,45]. In accordance, our data reveals that the majority of DEGs related to proteolytic processes are upregulated in muscle of cachectic OLCC mice and belong to catabolic pathways, with E3 Ub-ligases including *Atrogin-1* and *Murf-1*, as well as *Map1lc3b*, *Bnip3 and Sqstm1*, as markers of UPS activation and ALP alterations, respectively. No differences regarding food intake were present in these mice, suggesting that these processes are not attributable to anorexia [38]. Activation of UPS and ALP have previously been reported in skeletal muscle of OLCC model mice [38], and are in line with observations in other preclinical CAC models [15,46]. However, the extent to which UPS activation occurs in the OLCC model (10–15-fold based on *Atrogin-1* and *Murf-1* expression) is much lower than in CAC models elicited by ectopic tumor implantation (e.g., C26 or LLC), and more in accordance with *Atrogin-1* and *Murf-1* levels observed in other orthotopic models such as the KPP model [47]. Importantly, UPS activation seems less pronounced in human CAC [47], while the upregulation of ALP-related gene expression observed in OLCC muscle in the current study corresponds to findings in muscles of lung cancer patients with cachexia [10]. As the kinetics of CAC in the KPP and OLCC models strongly differ from those reported in many subcutaneous (ectopic) tumor implantation models [48], the slower progression of muscle mass loss in the orthotopic models may involve processes that better reflect the pathophysiological mechanisms in skeletal muscle of patients with CAC.

In contrast to the findings in atrophying muscles in OLCC mice, skeletal myotube cultures incubated with conditioned media of CAC-inducing 344P tumor cells show no signs of atrophy, i.e., no visually detected changes in myotube size. Accordingly, the expression of key regulatory genes involved in UPS (*Murf-1* and *Atrogin-1*), ALP (*Map1lc3b*) and protein synthesis (*DDIT4*) is not significantly increased by tCM. This seems to contradict various studies in which tCM prepared from in vivo cachexia-inducing tumors elicit catabolic signaling in cultured myotubes [49,50]. However, actual proteolysis measurements are often not included in these studies, and even with the use of the same tumor cells as a source, inconsistencies in activation of catabolic signaling have been reported between studies, e.g., C26 [51,52] and LLC [53,54] tumor cells. The latter may also reflect differences in tCM exposure regimes, which may be critical to observing muscle atrophy responses [55]. In some studies, appropriate controls like non-cachectic cell lines are missing [49,50]. This suggests the possibility of detecting atrophy-associated signaling in tCM-exposed myotubes resulting from media substrate depletion by the tumor cells, rather than induced by the tumor secretome. In our study this risk was mitigated by including the non-cachectic C10 lung epithelial cell line as a control condition. Discrepancies may also be attributable to differences in the tissue origin of the tumor, e.g., colon for C26 and lung for 344P, or the presence or absence of specific mutations that may drive specific cachexia subtypes. Indeed, another study [53] also reported a lack of myotube atrophy in response to tCM obtained from lung tumor cells carrying the KrasG12D mutation, which elicit cachexia and muscle atrophy in vivo, similarly to our findings with 344P lung tumor cells. Considering the absence of myotube atrophy and catabolic signaling following exposure to 344P tCM, the activation of the proteolysis machinery in skeletal muscle of OLCC mice is unlikely to reflect a direct response to tumor-derived factors but suggests a host-dependent mechanism. This may rely on tumor interactions with other tissues, e.g., liver [56] or immune cells [57], which can cause alterations in their metabolic or secretory behavior, or activation of the HPA axis, resulting in release of cortisol and activation of proteolysis-associated gene expression [58]. In line with this notion, glucocorticoid receptor-mediated gene expression is increased in OLCC muscle [38]. Alternatively, muscle proteolysis may be driven by a starvation-like metabolic state resulting from anorexic effects induced by GDF15 [59]. The absence of tCM effects on muscle proteolysis does not rule out the potential involvement of GDF15, as its cognate receptor GFRAL is not expressed in skeletal muscle [60], and its muscle proteolysis effects appear mostly CNS-mediated. While this study did not address potential involvement of altered protein synthesis, follow-up work using cancer cachexia models, in which reduced muscle protein synthesis rates are confirmed, could benefit from a parallel in vitro and in vivo comparison as deployed here. This will enable the dissection of tumor- and host-dependent alterations in muscle protein synthesis in cancer cachexia. Combined, our observations of the activation of muscle proteolytic pathways by 344P lung tumor cells only in vivo suggest involvement of various interconnected processes within the host environment, rather than a direct tumor-mediated effect.

RNA-seq analysis from hindlimb muscles of OLCC mice shows differential regulation of multiple myogenic processes. Although GO analysis indicates both up- and downregulated processes, the literature-curated 56-gene myogenesis set shows strong coordinated downregulation, driven by master regulators *MyoD1* and *Pax7*, with *Myostatin* as the only upregulated signaling gene. Our findings are suggestive of potential impairments in muscle regeneration. In line with this, disrupted myogenesis and muscle regenerative capacity have been described in atrophying muscle in CAC [61,62,63,64].

Following satellite cell activation and myoblast proliferation, *Pax7* expression levels decrease and the expression of the early myogenic markers *MyoD* and *Myogenin* increases, activating the myogenic differentiation program, including fusion and expression of muscle-specific genes, i.e., MyHC [65,66]. In our study *Pax7*, *MyoD*, *Myogenin* and MyHC (neonatal, *MYH8*; type I, *MYH7*; type IIb, *MYH4*) mRNA levels are regulated accordingly in differentiating C2C12, but when exposed to tCM, these expression patterns are strongly disrupted. Failure to reduce *Pax7* levels under differentiation conditions in myoblasts exposed to tCM corresponds to findings demonstrating persistent expression of *Pax7* in CAC muscle, which interferes with the regenerative capacity of myogenic cells [24], and other studies linking aberrant *Pax7* regulation to impaired muscle regeneration [67] and fusion defects [24]. Accordingly, *Myomaker* expression levels are also suppressed by tCM, and in line with its postulated role in myogenic cell fusion [68], myotube formation and myonuclear accretion quantified in our postnatal fusion analysis, are strongly reduced in presence of tCM. Although the underlying mechanisms of impaired myogenic fusion, e.g., dysregulation of other regulatory molecules like *Myomerger*, remain to be explored, overall coordinated inhibition of myogenic differentiation in response to tCM has also been reported by others [69]. However, as starvation may inhibit myogenic differentiation via cell-cycle inhibition [55,70], we now rule out substrate depletion as a cause of compromised myogenesis, as glucose supplementation does not reverse the impairing effects of tCM. Combined, our data demonstrates evidence that inhibition of myogenic differentiation is a phenotype induced by 344P tumor cells that is preserved in vivo and in vitro, suggesting impaired myogenesis is a direct, tumor-mediated response that may contribute to muscle wasting in CAC. Although multiple factors have been proposed to mediate muscle wasting in CAC, the tumor-secreted mediators responsible for impaired myogenesis remain to be identified [69].

The metabolic derangements in CAC have been postulated to result from muscle mitochondrial dysfunction [71,72,73]. Accordingly, our RNAseq analysis reveals profound downregulation of mitochondria-related pathways, with OXPHOS subunits and assembly factors most affected in atrophying muscle of OLCC mice. Both nuclear and mitochondria-encoded transcripts are reduced, and mitochondrial DNA copy number is decreased, in OLCC muscle, implying coordinated regulation at these levels, in line with previous studies [74,75]. However, protein levels of OXPHOS complexes in the muscles are not different between OLCC and sham mice. Interestingly, this discordance in regulation of mRNA levels and protein abundance [76] of OXPHOS complexes is also observed in skeletal muscle of cachectic NSCLC patients. Sustained OXPHOS protein levels in CAC may not equal preserved mitochondrial function, as mitochondrial respiration was strongly reduced in skeletal muscle of LLC-induced CAC mice despite conserved COXIV abundance [77]. While the disrupted mitochondrial homeostasis implicated by the transcriptomic data and by the discordant mtDNA and OXPHOS protein abundance remains to be confirmed by functional measurements, the strong induction of UCP3 expression levels may point to impaired mitochondrial function in skeletal muscle of OLCC mice. Moreover, this elevation is also in line with the reported increases in hydrogen peroxide production in CAC muscle [77], as induction of UCP3 may reflect an adaptive response to suppress mitochondria-derived oxidative stress [78,79].

To separate direct tumor-induced effects from host-dependent mechanisms responsible for the mitochondrial dysfunction implied by the OLCC muscle transcriptomic analyses, tumor-conditioned media were applied to muscle cell cultures. Mitochondrial respiration is reduced in C2C12 myotubes exposed to 344P tCM, in line with other studies deploying cachexia-inducing tCM [70,80]. As spare respiratory capacity specifically is affected, this suggests changes in substrate transport or oxidation [81]. Decreases in SRC have been linked to glycolytic adjustments [82], which is consistent with our observations revealing increases in the expression of various glycolytic genes in cultured myotubes after tCM-exposure. Our findings are in line with a shift towards glycolytic dominance demonstrated at mRNA transcript and enzymatic activity levels in muscle cells exposed to cachexia-inducing colon tCM [83]. Increased reliance on glycolysis may reflect an adaptive response to maintain energy homeostasis. Accordingly, cellular ATP content is maintained in myotube cultures in presence of tCM. However, longer-term exposure to tumor-derived factors may elicit such changes, as a reduction in ATP production has previously been reported in CAC muscle [84]. Counterintuitively, CS enzymatic activity increases in response to 344P tCM, but as it does not culminate in an effective increase in oxidative phosphorylation (i.e., increase in maximal respiration and ATP content), this may reflect a futile adaptive response, or complex, potentially maladaptive mitochondrial remodeling. Combined, these results indicate that while not all measurements of mitochondrial function are equally affected, the coordination of mitochondria-related processes is disrupted in muscle cell cultures exposed to 344P tCM as well as skeletal muscle of 344P lung tumor-bearing cachectic mice. Similar heterogeneous mitochondrial alterations have been described in skeletal muscle of preclinical CAC models and postulated to precede skeletal muscle wasting [85]. Finally, further aligning our in vitro and in vivo findings, muscle mtDNA content is reduced in tCM-exposed muscle cell cultures as well in OLCC muscle, indicating that mitochondrial dysfunction is a direct effect elicited by tumor-secreted mediators.

The causal involvement of mitochondrial dysfunction in muscle atrophy in CAC is still debated [86], and our study design in the OLCC model was not set up to address this directly. However, comparison with the parallel in vitro experiments infers that muscle catabolic signaling in OLCC mice is unlikely to result from tumor-induced mitochondrial dysfunction, as no activation of proteolysis is observed in response to tCM or following CCCP incubation. In line with our findings, myotubes exposed to tCM-derived vesicles (TMVs) displayed mitochondrial dysfunction in absence of any atrophy [70]. Moreover, the direct induction of mitochondrial dysfunction with CCCP in myotubes does not activate any of the common proteolytic pathways associated with CAC-associated muscle wasting. Instead, induction of *REDD1* is present, which may suggest inhibition of protein synthetic pathways, and reflect a mechanism to preserve energy [83,87].

Besides supporting muscle protein synthesis, mitochondrial function is essential for maintenance of skeletal muscle mass by facilitating myogenic differentiation [88]. Both mitochondrial function and myogenesis are affected in muscles of OLCC mice and in cultured skeletal muscle cells exposed to tCM. In response to TMVs prepared from colon tumor cells, a similar delay in myogenic differentiation of C2C12 myoblasts was reported in presence of reduction in various indices of mitochondrial function [70]. This was accompanied by suppression of mitochondrial biogenesis, a process intricately involved in myogenesis [89]. In mitochondria, coupling efficiency of oxygen consumption and oxidative phosphorylation is governed by uncoupling proteins, including UCP3 in muscle specifically [78]. To investigate the importance of UCP3 upregulation, which is the only mitochondria-related gene increased in cachectic OLCC muscle, cultured muscle cells were incubated with CCCP to emulate UCP-evoked mitochondrial dysfunction. Although CCCP induces less specific protein leaking than UCP3, the majority of metabolic responses to tCM and CCCP overlap, and importantly, postnatal myogenesis is similarly impaired by CCCP and tCM. Combined, these data suggest that mitochondrial dysfunction contributes to impaired myogenesis. Although some evidence supports modulating mitochondrial function as a viable strategy to target myogenesis and improve muscle regeneration in tumor-bearing mice [90], it remains to be explored whether this will attenuate CAC-associated muscle atrophy in a relevant preclinical setting like the OLCC model. Moreover, Beltra et al. postulate that mitochondria-targeted anti-cachexia interventions mostly influence protein degradation and synthesis [91]. Consequently, the interrelationships between mitochondrial function, protein turnover and myogenesis in CAC require further investigation. Nevertheless, the systematic dissection of their tumor- and host-dependent dysregulation is essential for determining the tissue origin and identification of the factors that control these processes. Subsequent inclusion of their assessment in routine blood analyses of patients at risk for cachexia, and targeted inhibitory treatment as a pharmacological component of a multimodal therapy, will enable personalized treatment of cachexia. This will improve clinical outcomes and cost-effectiveness of NSCLC surgical [92] and non-resectable anti-tumor treatment therapies, and reduce overall disease burden for patients.

## 5. Conclusions

Atrophying skeletal muscle in lung cancer cachexia is characterized by increased proteolysis signaling, impaired myogenesis and mitochondrial deficiencies. Factors present in the cachexia-inducing lung tumor secretome directly impair myogenesis and muscle mitochondrial function, whereas activation of muscle catabolic processes relies on host-driven mechanisms.

## Data Availability

All experimental data generated or analyzed during this study are included in this published article and its additional information files. The RNA sequencing data generated in this study have been deposited in Zenodo (https://doi.org/10.5281/zenodo.19555914). The bioinformatics analysis pipeline and scripts are available at Zenodo. MitoCarta 3.0 data were obtained from the Broad Institute (https://www.broadinstitute.org/mitocarta, accessed on 12 November 2025). Gene Ontology annotations were retrieved from the QuickGO REST API (https://www.ebi.ac.uk/QuickGO/, accessed on 13 April 2026). The GO ontology structure (go-basic.obo, December 2025 release) was obtained from the Gene Ontology Consortium (https://geneontology.org/).

## Supplementary Materials

The following supporting information can be downloaded at: https://www.mdpi.com/article/10.3390/cancers18132130/s1. Figure S1: Body weight, muscle mass and -function in CAC mice compared to healthy sham mice included in transcriptomic analysis. Relative change in percentage of total body weight at the end of the experiment (A). Absolute change in grams of forearm grip strength compared to baseline (B). Absolute change in CT-derived muscle mass compared to baseline (C). Relative change in percentage of total muscle mass normalized to total body weight at baseline (D). Data is presented as mean ± SEM. Significance is shown as * *p* ≤ 0.05. Figure S2: Genome-wide enrichment landscape of the cachectic muscle transcriptome. Specific GO Biological Process terms over-represented among all DEGs (g:Profiler, top 15 non-redundant terms of intermediate size, bar length −log10 FDR, in-bar number = DEGs in term) (A). GO Biological Process gene sets most enriched by pre-ranked GSEA of all expressed genes ranked by the signed DESeq2 statistic, red = enriched among up-regulated genes, blue = enriched among down-regulated genes (bar length = normalised enrichment score) (B). Figure S3: Upstream regulators of the proteolysis and myogenesis programmes. Combined directed network of the top ten enriched transcription factors (TF) of proteolysis and myogenesis (union: 17 TFs [squares], fill colour = log2 fold-change of the TF’s own transcript, grey if not detected in the RNA-seq and the proteolysis [circles] and myogenesis [diamonds] DEGs they target. Blue indicates downregulated, red indicates upregulated. A green border on a TF indicates it is itself a DEG. Figure S4: Myotube atrophy in C2C12 myotubes. Phase contrast images of C2C12 myotubes (d8) after 3 day exposure to DM, cCM or tCM (A). Phase contrast images of C2C12 myotubes exposed to vehicle control (VC; 0.125% DMSO) or DEX treatment (75 μM) after 24 h (B). Fold changes in mRNA expression levels after DEX treatment after 8 and 24 h compared to VC of Atrogin-1 (C) and Murf-1 (D). Nested T-test (B). * *p*-value ≤ 0.05, *** *p*-value ≤ 0.001. Figure S5: Postnatal fusion after 393p conditioned medium exposure in C2C12. C2C12 myoblasts and myotubes exposed to DM, 344P CM (tCM) or 393P CM. Data is presented as mean ± SD. Significance is shown as **** *p*-value ≤ 0.0001. Figure S6: Three-panel analysis of MitoCarta 3.0 pathway annotations showing differential expression patterns across 105 mitochondrial pathways organized in three hierarchical levels. Color scheme: Blue indicates downregulated genes, red indicates upregulated genes. A: Top-level functional categories showing overall mitochondrial pathway regulation. Metabolism, OXPHOS, and Mitochondrial central dogma represent the most affected categories. Protein import, sorting, and homeostasis and Small molecule transport also show significant directional bias (A). Intermediate pathways showing more granular functional categories. Translation, Metals and cofactors, and mtRNA metabolism show the strongest downregulation. OXPHOS subunits and assembly factors, along with Complex I, IV, and V demonstrate complete suppression of oxidative phosphorylation machinery (B). Most granular pathway annotations showing specific functional subcategories. CI subunits, CIV assembly factors, and CI assembly factors represent the most significantly affected detailed pathways. The pattern of coordinated downregulation is consistent across all levels (C). Figure S7: Uncropped Western Blots of OXPHOS complexes. Complex I (20 kD), Complex II (30 kD), III (48 kD) and V (55 kD). Blot B has been cut from blot A for a longer exposure time (A,B). Correction by total protein content via PonceauS (C). Figure S8: Oxygen consumption rate (OCR) values measured via Seahorse XF. Protein-normalized OCR values (A). Total protein content according to Pierce BCA protein assay used for normalization of oxygen consumption rates (B). Final basal values used for percentual OCR calculations in Figure 7 (C). Maximal respiration and non-mitochondrial respiration corrected for protein (D,E). Coupling efficiency calculated as (ATP-related / basal respiration) x 100 (F). ** *p*-value ≤ 0.01; *** *p*-value ≤ 0.005. Figure S9: Catabolic markers in C2C12 myotubes after mitochondrial inhibitor CCCP exposure. Gene expression levels of regulatory molecules of protein turnover after CCCP exposure for 8, 16 and 24 h (A,B). * *p*-value ≤ 0.05. Table S1: Primer list qPCR. Table S2: Concentrations of compounds used during Seahorse XF Cell Mito Stress test. Table S3: DEG related to proteolysis (*n* = 21). Table S4: Manual literature-based search for myogenic regulators (*n* = 56).
