# Supplementary material for: The Secretome of a Cachexia-Inducing Lung Tumor Impairs Mitochondrial Function and Skeletal Muscle Differentiation
Source: Cancers (Basel). 2026 Jun 30;18(13):2130. doi: 10.3390/cancers18132130 (PMC13359459; doi:10.3390/cancers18132130)
Supplement: Supplementary file 1 [file cancers-18-02130-s001.zip › Supplementary figures.pdf]

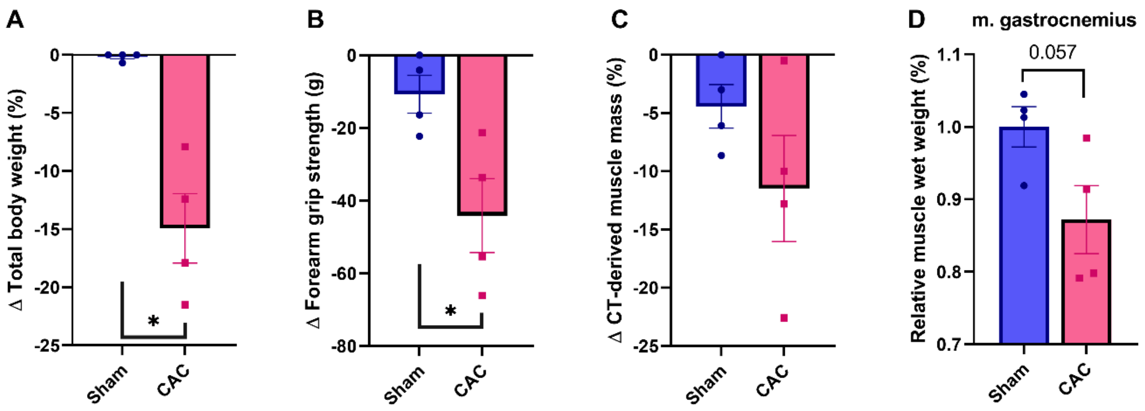


**Figure S1: Body weight, muscle mass and -function in CAC mice compared to healthy sham mice included in transcriptomic analysis.** Relative change in percentage of total body weight at the end of the experiment (A). Absolute change in grams of forearm grip strength compared to baseline (B). Absolute change in CT-derived muscle mass compared to baseline (C). Relative change in percentage of total muscle mass normalized to total body weight at baseline (D). Data is presented as mean ± SEM. Significance is shown as $*P \leq 0.05$.

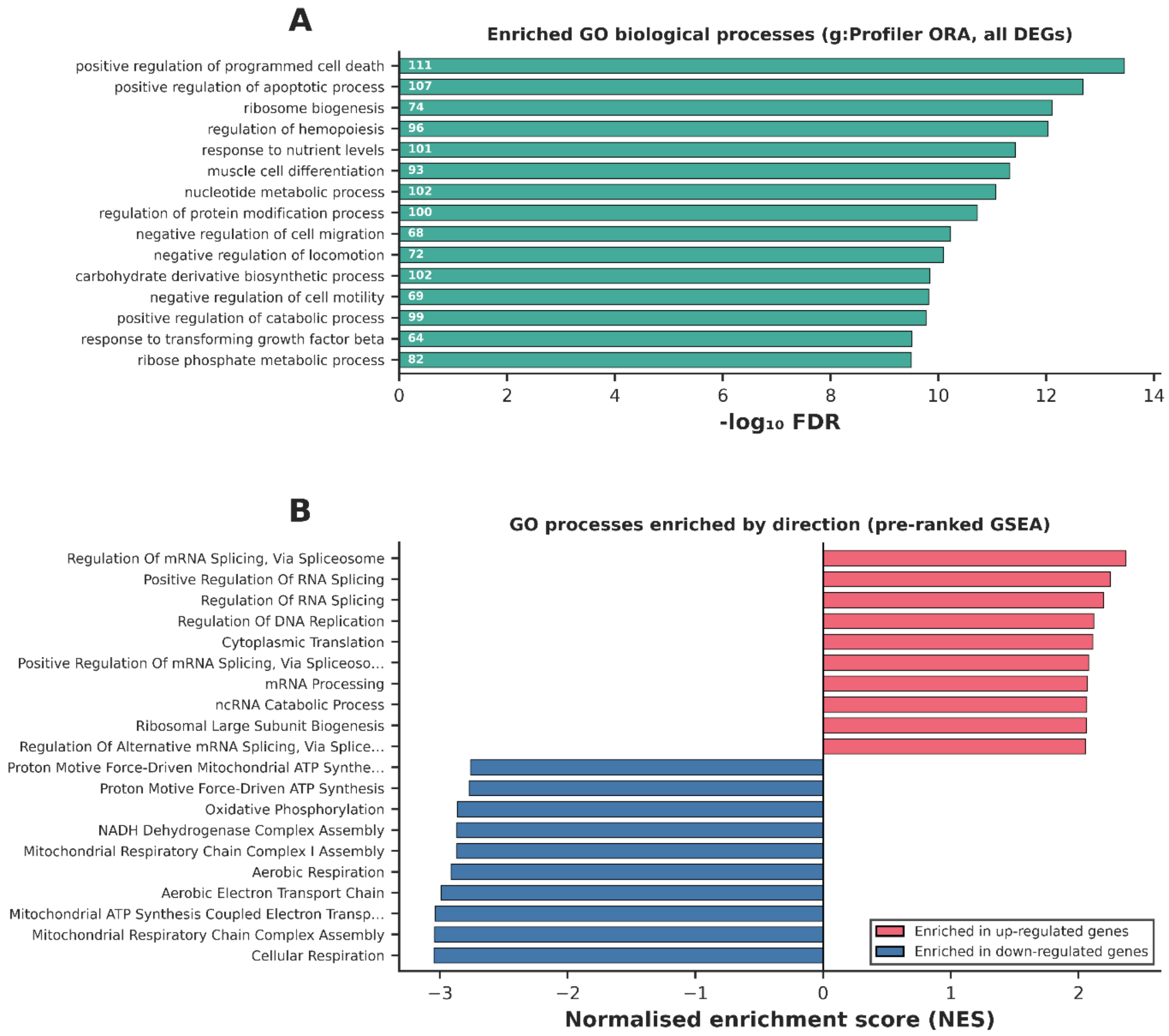


**Figure S2: Genome-wide enrichment landscape of the cachectic muscle transcriptome.** Specific GO Biological Process terms over-represented among all DEGs (g:Profiler, top 15 non-redundant terms of intermediate size, bar length −log10 FDR, in-bar number = DEGs in term) (A). GO Biological Process gene sets most enriched by pre-ranked GSEA of all expressed genes ranked by the signed DESeq2 statistic, red = enriched among up-regulated genes, blue = enriched among down-regulated genes (bar length = normalised enrichment score) (B).

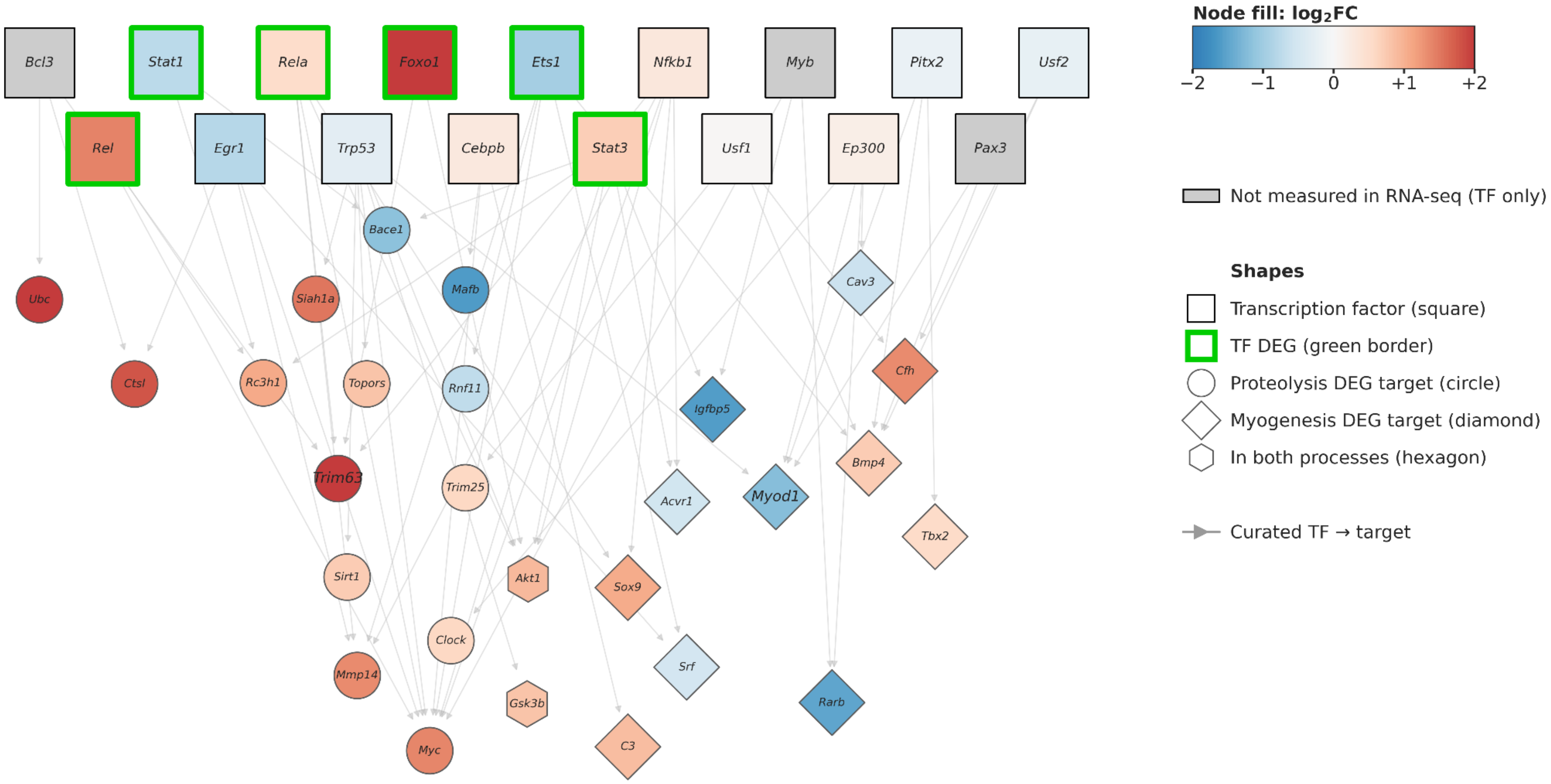


**Figure S3: Upstream regulators of the proteolysis and myogenesis programmes.** Combined directed network of the top ten enriched transcription factors (TF) of proteolysis and myogenesis (union: 17 TFs [squares], fill colour = log2 fold-change of the TF's own transcript, grey if not detected in the RNA-seq and the proteolysis [circles] and myogenesis [diamonds] DEGs they target. Blue indicates downregulated, red indicates upregulated. A green border on a TF indicates it is itself a DEG.

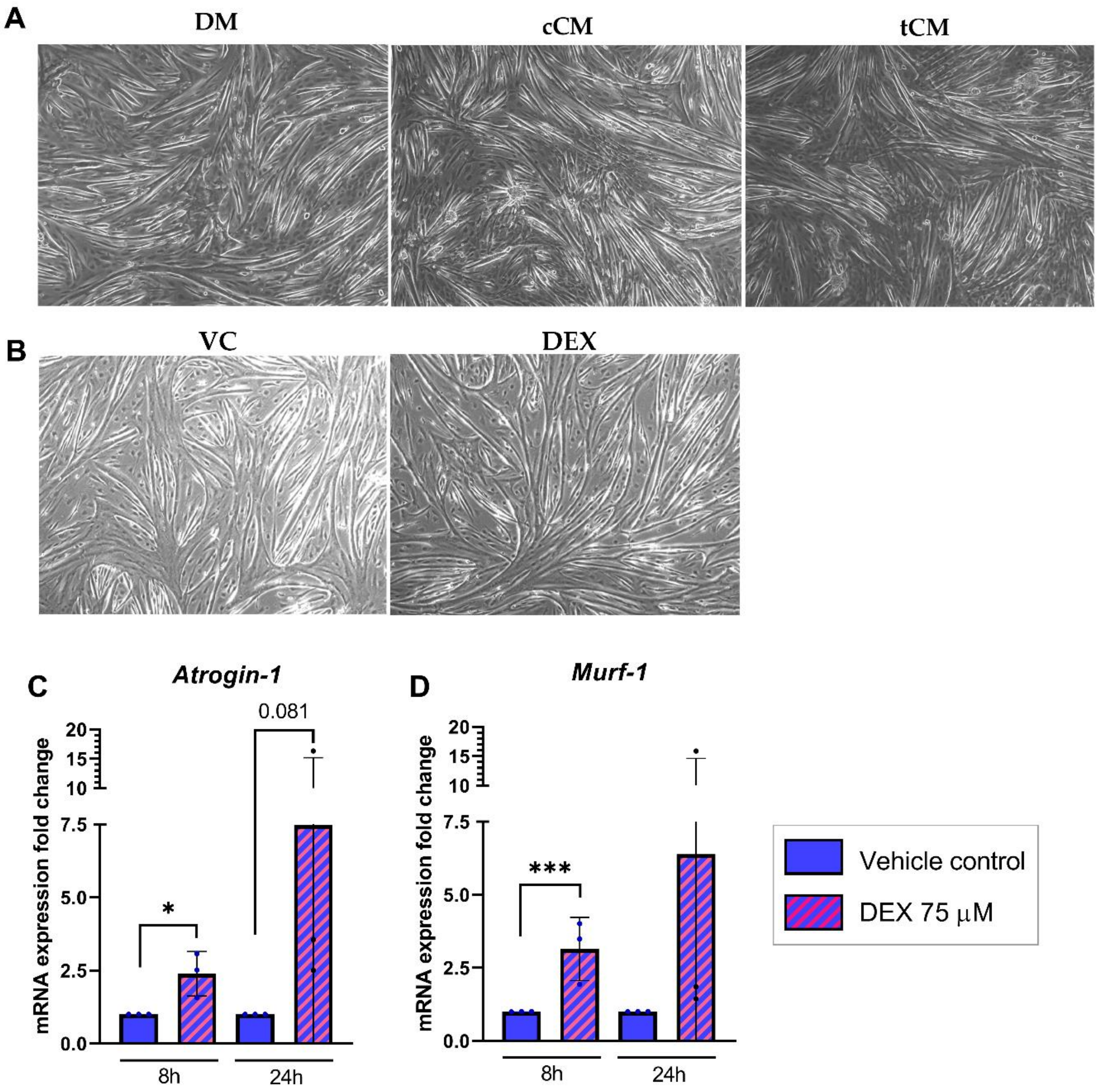


**Figure S4: Myotube atrophy in C2C12 myotubes.** Phase contrast images of C2C12 myotubes (d8) after 3 day exposure to DM, cCM or tCM (A). Phase contrast images of C2C12 myotubes exposed to vehicle control (VC; 0.125% DMSO) or DEX treatment (75 μM) after 24 hours (B). Fold changes in mRNA expression levels after DEX treatment after 8 and 24 hours compared to VC of Atrogin-1 (C) and Murf-1 (D). Nested T-test (B). *P-value≤0.05, ***P-value≤0.001.

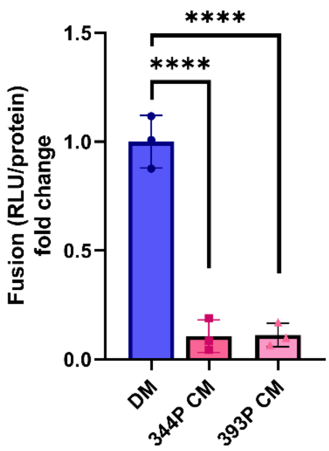


**Figure S5: Postnatal fusion after 393p conditioned medium exposure in C2C12.** C2C12 myoblasts and myotubes exposed to DM, 344P CM (tCM) or 393P CM. Data is presented as mean ± SD. Significance is shown as ****P-value≤0.0001.

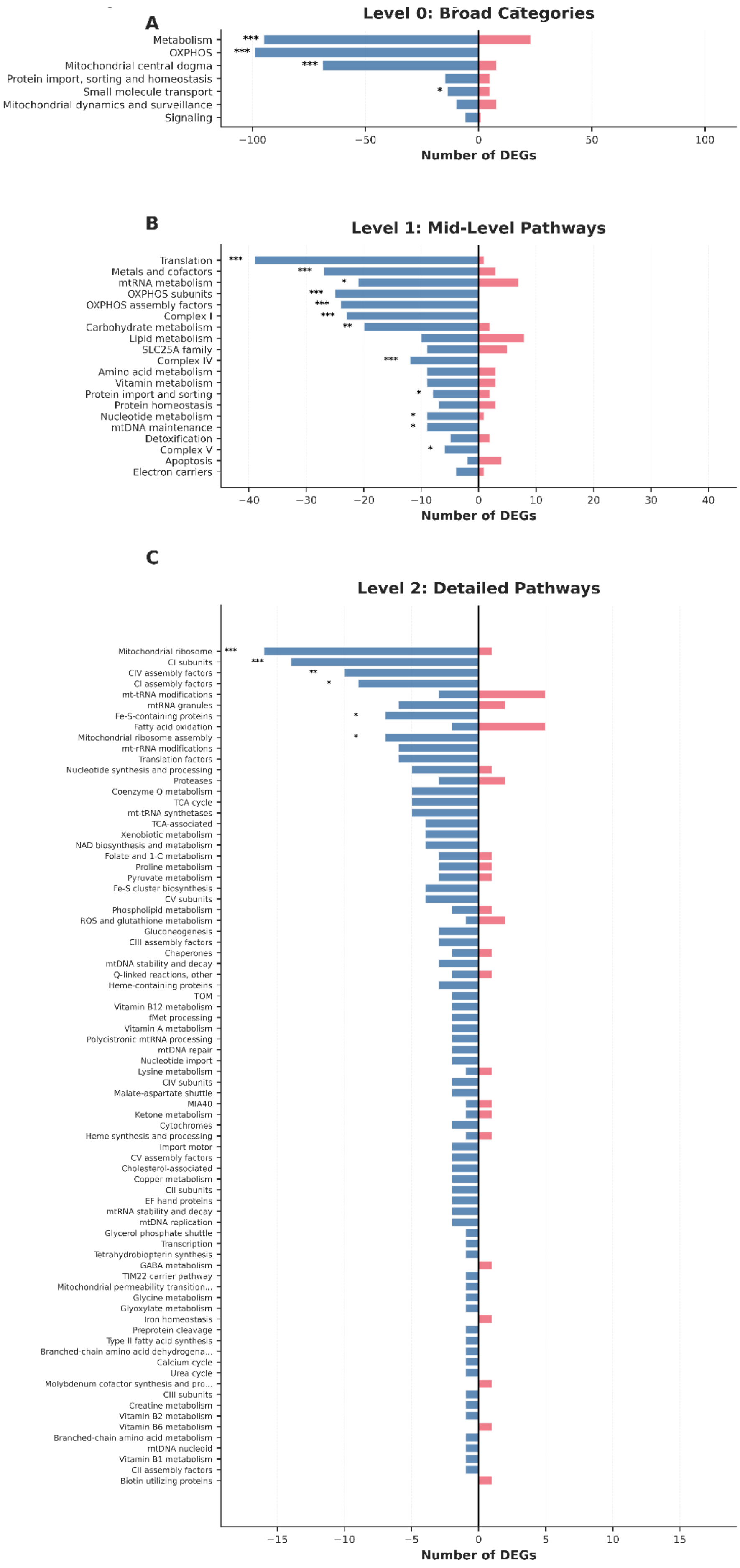
A
Level 0: Broad Categories
Metabolism
OXPHOS
Mitochondrial central dogma
Protein import, sorting and homeostasis
Small molecule transport
Mitochondrial dynamics and surveillance
Signaling
***
***
***
*
−100
−50
0
50
100
Number of DEGs
B
Level 1: Mid-Level Pathways
Translation
Metals and cofactors
mtRNA metabolism
OXPHOS subunits
OXPHOS assembly factors
Complex I
Carbohydrate metabolism
Lipid metabolism
SLC25A family
Complex IV
Amino acid metabolism
Vitamin metabolism
Protein import and sorting
Protein homeostasis
Nucleotide metabolism
mtDNA maintenance
Detoxification
Complex V
Apoptosis
Electron carriers
***
***
*
***
***
***
**
***
*
*
*
*
−40
−30
−20
−10
0
10
20
30
40
Number of DEGs
C
Level 2: Detailed Pathways
Mitochondrial ribosome
CI subunits
CIV assembly factors
CI assembly factors
mt-tRNA modifications
mtRNA granules
Fe-S-containing proteins
Fatty acid oxidation
Mitochondrial ribosome assembly
mt-rRNA modifications
Translation factors
Nucleotide synthesis and processing
Proteases
Coenzyme Q metabolism
TCA cycle
mt-tRNA synthetases
TCA-associated
Xenobiotic metabolism
NAD biosynthesis and metabolism
Folate and 1-C metabolism
Proline metabolism
Pyruvate metabolism
Fe-S cluster biosynthesis
CV subunits
Phospholipid metabolism
ROS and glutathione metabolism
Gluconeogenesis
CIII assembly factors
Chaperones
mtDNA stability and decay
Q-linked reactions, other
Heme-containing proteins
TOM
Vitamin B12 metabolism
fMet processing
Vitamin A metabolism
Polycistronic mtRNA processing
mtDNA repair
Nucleotide import
Lysine metabolism
CIV subunits
Malate-aspartate shuttle
MIA40
Ketone metabolism
Cytochromes
Heme synthesis and processing
Import motor
CV assembly factors
Cholesterol-associated
Copper metabolism
CII subunits
EF hand proteins
mtRNA stability and decay
mtDNA replication
Glycerol phosphate shuttle
Transcription
Tetrahydrobiopterin synthesis
GABA metabolism
TIM22 carrier pathway
Mitochondrial permeability transition...
Glycine metabolism
Glyoxylate metabolism
Iron homeostasis
Preprotein cleavage
Type II fatty acid synthesis
Branched-chain amino acid dehydrogena...
Calcium cycle
Urea cycle
Molybdenum cofactor synthesis and pro...
CIII subunits
Creatine metabolism
Vitamin B2 metabolism
Vitamin B6 metabolism
Branched-chain amino acid metabolism
mtDNA nucleoid
Vitamin B1 metabolism
CII assembly factors
Biotin utilizing proteins
***
***
**
*
*
*
−15
−10
−5
0
5
10
15
Number of DEGs

**Figure S6: Three-panel analysis of MitoCarta 3.0 pathway annotations showing differential expression patterns across 105 mitochondrial pathways organized in three hierarchical levels.** Color scheme: Blue indicates downregulated genes, red indicates upregulated genes. A: Top-level functional categories showing overall mitochondrial pathway regulation. Metabolism, OXPHOS, and Mitochondrial central dogma represent the most affected categories. Protein import, sorting, and homeostasis and Small molecule transport also show significant directional bias (A). Intermediate pathways showing more granular functional categories. Translation, Metals and cofactors, and mtRNA metabolism show the strongest downregulation. OXPHOS subunits and assembly factors, along with Complex I, IV, and V demonstrate complete suppression of oxidative phosphorylation machinery (B). Most granular pathway annotations showing specific functional subcategories. CI subunits, CIV assembly factors, and CI assembly factors represent the most significantly affected detailed pathways. The pattern of coordinated downregulation is consistent across all levels (C).

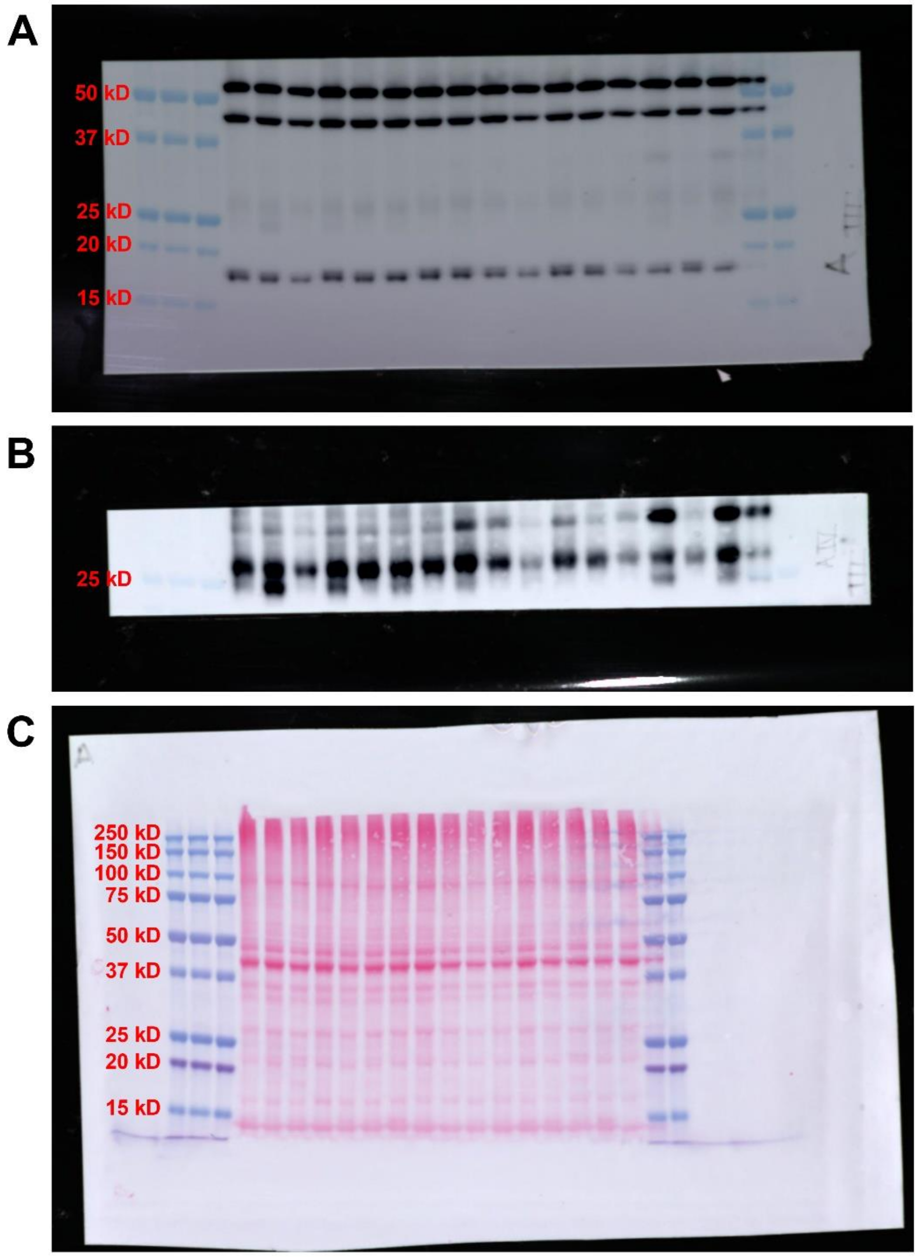


**Figure S7: Uncropped Western Blots of OXPHOS complexes.** Complex I (20 kD), Complex II (30 kD), III (48 kD) and V (55 kD). Blot B has been cut from blot A for a longer exposure time (A-B). Correction by total protein content via PonceauS (C).

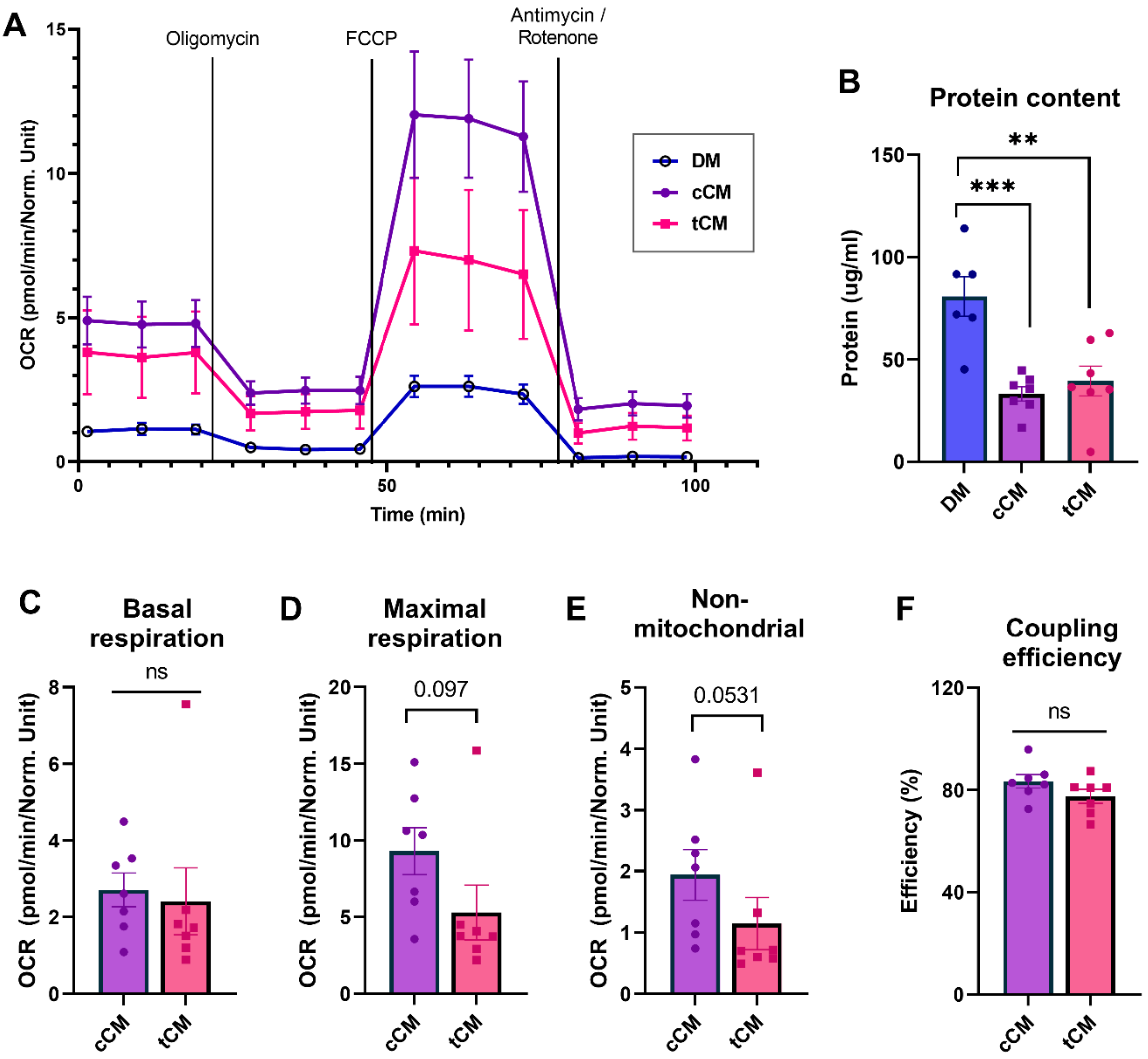


**Figure S8: Oxygen consumption rate (OCR) values measured via Seahorse XF.** Protein-normalized OCR values (A). Total protein content according to Pierce BCA protein assay used for normalization of oxygen consumption rates (B). Final basal values used for percentual OCR calculations in Figure 7 (C). Maximal respiration and non-mitochondrial respiration corrected for protein (D, E). Coupling efficiency calculated as (*ATP-related / basal respiration) x 100* (F). **P-value≤0.01; ***P-value≤0.005.

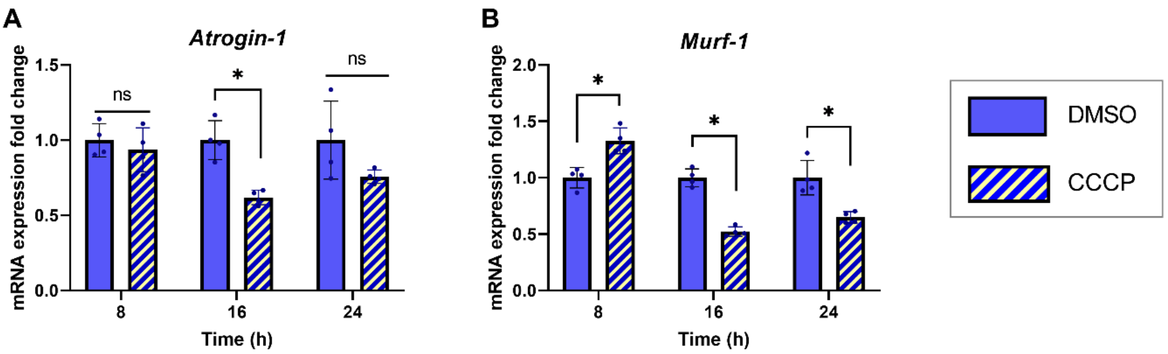


**Figure S9: Catabolic markers in C2C12 myotubes after mitochondrial inhibitor CCCP exposure.** Gene expression levels of regulatory molecules of protein turnover after CCCP exposure for 8, 16 and 24 hours (A-B). *P-value≤0.05.
