# Supplementary material for: The Secretome of a Cachexia-Inducing Lung Tumor Impairs Mitochondrial Function and Skeletal Muscle Differentiation
Source: Cancers (Basel). 2026 Jun 30;18(13):2130. doi: 10.3390/cancers18132130 (PMC13359459; doi:10.3390/cancers18132130)
Supplement: Supplementary file 1 [file cancers-18-02130-s001.zip › Supplementary tables.pdf]

Table S1: Primer list qPCR

Target	Sense primer	Antisense primer
**Atrogin-1**	CAGCAGCTGAATAGCATCCAGAT	TCTGCATGATGTTCAGTTGTAAGC
**MURF-1**	CTTCCTCTCAAGTGCCAAGCA	GTGTTCTAAGTCCAGAGTAAAGTAGTCCAT
**REDD1 (Ddit4)**	TCGGCGCTTCACTACTGACC	CCTAACACCCACCCCATTCC
**LC3b**	GAGCAGCACCCCACCAAGAT	CGTGGTCAGGCACCAGGAA
**Pax7**	ACCAGTACAGCCAGTATGGCCA	GTGTTCCCCAAGCTTCATACGG
**Myf5**	GAACAGCAGCTTTGACAGCAT	AATGCTGGACAAGCATCCAA
**MyoD**	GGCCGTGGCAGCGAG	CGCTGTAATCCATCATGCCAT
**Myogenin**	CCCATGGTGCCCAGTGAA	GCAGATTGTGGGCGTCTGTA
**Myomaker**	CTGGCCGACTTTGATGAACC	TGCTCTTGTCGGGGTACAGG
**MHC IIB (MYH4)**	ACAAGCTGCGGGTGAAGAGC	CAGGACAGTGACAAAGAACG
**MHC I (MYH7)**	CAGATCGGGAGAATCAGTCCAT	AGCAAAATATTGGATGACCCTCTTA
**MHC neonatal (MYH8)**	ACACATCTTGCAGAGGAAGG	TAAACCCAGAGAGGCAAGTG
**MCK**	AGGTTTTCCGCCGCTTCT	CGGTGCCCAGGTTGGA
**COXII**	CCATCCCAGGCCGACTAA	ATTTCAGAGCATTGGCCATAGAA
**ND1**	CAGGATGAGCCTCAAACTCC	GGTCAGGCTGGCAGAAGTAA
**NRF1**	AGCCACATTGGCTGATGCTT	GGTCATTTCACCGCCCTGTA
**PGC1α**	CAACAATGAGCCTGCGAACA	CTTCATCCACGGGGAGACTG
**TFAM**	CCGGCAGAGACGGTTAAAAA	TCATCCTTTGCCTCCTGGAA
**HKII**	AACCTCAAAGTGACGGTGGGC	AAGGACACGTCACATTTCGGAGC
**PFK1A**	GCGATCTCCAGGTGAATGTT	CACGTTCTTCCTGCTGTCAA
**PFKFB3**	AGAACTTCCACTCTCCCACCCAAA	AGGGTAGTGCCCATTGTTGAAGGA
**PDK4**	AGGTTATGGGACAGACGCTATCAT CTACT	AGACTGGGAGCTTTTCTACAGACTCAGA
**GLUT1**	TGACCATCGCCCTGGCCT	GGACCAGGGCCTACTTCAAAGAAG
**FGF21**	CAAGCATACCCCATCCCTGA	TGGCTGTTGGCAAAGAAACC
**CycloA**	TTCCTCCTTTCACAGAATTATTCCA	CCGCCAGTGCCATTATGG
**Rplp0**	GGACCCGAGAAGACCTCCTT	GCACATCACTCAGAATTTCAATGG
**HPRT**	TGGATATGCCCTTGACTATAATGA GTAC	AGGACTCCTCGTATTTGCAGATTC
**GUSB**	CATTAGCAAGCTGGTCCAGAGT	GACAAAGTAACCCTTGGGATACAT
**B2M**	CTTTCTGGTGCTTGTCTCACTGA	GTATGTTCGGCTTCCCATTCTC

Table S2: Concentrations of compounds used during Seahorse XF Cell Mito Stress test

Compound	Stock concentration [mM]	Working concentration [uM]
**Oligomycin**	10	1
**FCCP**	40	4
**Rotenone / Antimycin A**	10 / 10	1 / 1

Table S3: DEG related to proteolysis (n=21).

Gene Symbol	Ensemble ID	baseMean	log2FC	Fold Change	pvalue	padj	Direction
**Trim63**	ENSMUSG00000028834	44995.10	3.74	14.31	1.98E-46	4.59E-43	↑
**Fbxo32**	ENSMUSG00000022358	49864.74	2.92	11.42	3.34E-30	1.49E-27	↑
**Cblb**	ENSMUSG00000022637	1532.52	1.96	8.45	2.80E-17	2.26E-15	↑
**Ulk1**	ENSMUSG00000029512	7775.60	1.51	6.60	3.98E-11	1.05E-09	↑
**Rnf4**	ENSMUSG00000029110	933.95	0.77	6.47	9.61E-11	2.38E-09	↑
**Gabarapl1**	ENSMUSG00000030161	3166.69	1.83	6.04	1.58E-09	2.96E-08	↑
**Rnf2**	ENSMUSG00000026484	737.28	0.61	5.26	1.47E-07	1.70E-06	↑
**Bnip3l**	ENSMUSG00000022051	2522.24	0.70	5.06	4.22E-07	4.30E-06	↑
**Psmd11**	ENSMUSG00000017428	5512.80	0.90	5.00	5.84E-07	5.73E-06	↑
**Map1lc3b**	ENSMUSG00000031812	8239.12	0.78	4.62	3.87E-06	3.04E-05	↑
**Bnip3**	ENSMUSG00000078566	10666.03	1.03	4.57	4.84E-06	3.68E-05	↑
**Sqstm1**	ENSMUSG00000015837	29359.88	0.90	4.53	6.04E-06	4.46E-05	↑
**Fbxo30**	ENSMUSG00000047648	795.70	1.28	4.36	1.31E-05	8.61E-05	↑
**Fbxo31**	ENSMUSG00000052934	9609.59	0.54	4.27	1.98E-05	0.000123	↑
**Psmd4**	ENSMUSG00000005625	6003.61	0.68	3.84	0.000122	0.000586	↑
**Psmd3**	ENSMUSG00000017221	5721.46	0.52	3.66	0.000255	0.001105	↑
**Psmc2**	ENSMUSG00000028932	4442.05	0.52	3.44	0.000581	0.00224	↑
**Psma7**	ENSMUSG00000027566	2806.91	0.52	3.01	0.002594	0.007954	↑
**Fundc1**	ENSMUSG00000025040	668.77	-0.60	-3.59	0.000336	0.001399	↓
**Trim32**	ENSMUSG00000051675	357.41	-0.60	-4.42	9.76E-06	6.74E-05	↓
**Psmb10**	ENSMUSG00000031897	177.45	-0.70	-4.97	6.84E-07	6.63E-06	↓

Table S4: Manual literature-based search for myogenic regulators (n=56).

Gene Symbol	Ensembl ID	Category	baseMean	log2FC	Fold Change	p-value	padj (FDR)	DEG (padj<0.01, ∣log2FC∣>=0.5)	Direction
**Pax7**	ENSMUSG00000028736	Myogenic Regulatory Factors	88.2	-1.361	2.57	4.52E-10	9.51E-09	**Yes**	↓
**Myod1**	ENSMUSG00000009471	Myogenic Regulatory Factors	535.7	-1.212	2.32	2.44E-07	2.66E-06	**Yes**	↓
**Myf6**	ENSMUSG00000035923	Myogenic Regulatory Factors	2165.5	-0.381	1.3	0.068	0.1203	No	ns
**Myog**	ENSMUSG00000026459	Myogenic Regulatory Factors	150.1	0.516	1.43	0.1513	0.2321	No	ns
**Myf5**		Myogenic Regulatory Factors						Not detected	
**Pax3**		Myogenic Regulatory Factors						Not detected	
**Myh8**	ENSMUSG00000055775	Myosin Heavy Chains	239.8	0.717	1.64	0.0089	0.0223	No	ns
**Myh1**	ENSMUSG00000056328	Myosin Heavy Chains	164910.5	0.392	1.31	0.0226	0.0487	No	ns
**Myh3**	ENSMUSG00000020908	Myosin Heavy Chains	230	0.645	1.56	0.0913	0.1535	No	ns
**Myh4**	ENSMUSG00000057003	Myosin Heavy Chains	3071566	0.11	1.08	0.4844	0.5876	No	ns
**Myh2**	ENSMUSG00000033196	Myosin Heavy Chains	103051.7	-0.172	1.13	0.5056	0.6071	No	ns
**Myh7**	ENSMUSG00000053093	Myosin Heavy Chains	36101.6	-0.252	1.19	0.6693	0.7479	No	ns
**Myl1**	ENSMUSG00000061816	Myosin Light Chains	162445.2	-0.752	1.68	7.08E-04	0.0026	**Yes**	↓
**Myl3**	ENSMUSG00000059741	Myosin Light Chains	4786.7	-0.547	1.46	0.2343	0.3299	No	ns
**Myl4**	ENSMUSG00000061086	Myosin Light Chains	143.2	0.287	1.22	0.2468	0.3433	No	ns
**Myl2**	ENSMUSG00000013936	Myosin Light Chains	4618.3	-0.277	1.21	0.5944	0.6852	No	ns
**Myl6**	ENSMUSG00000090841	Myosin Light Chains	2292.7	-0.065	1.05	0.6597	0.7398	No	ns
**Actg1**	ENSMUSG00000062825	Actin & Troponin Complex	8103.2	-0.754	1.69	6.44E-05	3.38E-04	**Yes**	↓
**Acta1**	ENSMUSG00000031972	Actin & Troponin Complex	953321.7	-0.464	1.38	4.86E-04	0.0019	No	ns
**Tpm1**	ENSMUSG00000032366	Actin & Troponin Complex	479463.7	-0.544	1.46	0.003	0.0089	**Yes**	↓
**Tnnc2**	ENSMUSG00000017300	Actin & Troponin Complex	192882.5	-0.538	1.45	0.003	0.0089	**Yes**	↓
**Actc1**	ENSMUSG00000068614	Actin & Troponin Complex	3634	-2.087	4.25	0.0044	0.0125	No	ns
**Tnnt2**	ENSMUSG00000026414	Actin & Troponin Complex	90.6	0.718	1.65	0.0076	0.0194	No	ns
**Tnnt3**	ENSMUSG00000061723	Actin & Troponin Complex	500949.8	-0.313	1.24	0.0277	0.0576	No	ns
**Tnni2**	ENSMUSG00000031097	Actin & Troponin Complex	179253.1	-0.321	1.25	0.0295	0.0607	No	ns

**Tpm2**	ENSMUSG00000028464	Actin & Troponin Complex	69212.8	-0.297	1.23	0.1993	0.2897	No	ns
**Tnnt1**	ENSMUSG00000064179	Actin & Troponin Complex	5004.4	-0.444	1.36	0.4008	0.5063	No	ns
**Tnnc1**	ENSMUSG00000091898	Actin & Troponin Complex	2706.6	-0.225	1.17	0.6838	0.7599	No	ns
**Tnni1**	ENSMUSG00000026418	Actin & Troponin Complex	2287.5	-0.209	1.16	0.7081	0.7799	No	ns
**Tpm3**	ENSMUSG00000027940	Actin & Troponin Complex	9244.6	-0.166	1.12	0.7242	0.7926	No	ns
**Tnni3**		Actin & Troponin Complex						Not detected	
**Myom1**	ENSMUSG00000024049	Sarcomere & Structural	70192.3	0.342	1.27	4.29E-04	0.0017	No	ns
**Myom2**	ENSMUSG00000031461	Sarcomere & Structural	107845.5	0.405	1.32	0.0043	0.0121	No	ns
**Neb**	ENSMUSG00000026950	Sarcomere & Structural	223053.3	0.153	1.11	0.0921	0.1546	No	ns
**Des**	ENSMUSG00000026208	Sarcomere & Structural	144144.6	0.103	1.07	0.1973	0.2875	No	ns
**Myom3**	ENSMUSG00000037139	Sarcomere & Structural	1825.3	-0.37	1.29	0.2438	0.3401	No	ns
**Vim**	ENSMUSG00000026728	Sarcomere & Structural	4684.3	0.138	1.1	0.4472	0.5519	No	ns
**Ttn**	ENSMUSG00000051747	Sarcomere & Structural	439952	-0.037	1.03	0.7052	0.7777	No	ns
**Vcam1**	ENSMUSG00000027962	Satellite Cell Markers	92.2	-0.951	1.93	1.35E-04	6.40E-04	**Yes**	↓
**Cd34**	ENSMUSG00000016494	Satellite Cell Markers	1465	-0.496	1.41	9.19E-04	0.0033	No	ns
**Itga7**	ENSMUSG00000025348	Satellite Cell Markers	3493.9	0.062	1.04	0.4433	0.5481	No	ns
**Cxcr4**		Satellite Cell Markers						Not detected	
**Gdf11**	ENSMUSG00000025352	Growth Factors & Signaling	458.6	-1.807	3.5	3.84E-09	6.50E-08	**Yes**	↓
**Mstn**	ENSMUSG00000026100	Growth Factors & Signaling	6601.2	0.606	1.52	2.29E-04	0.001	**Yes**	↑
**Fst**	ENSMUSG00000021765	Growth Factors & Signaling	291.7	0.962	1.95	0.0039	0.0112	No	ns
**Igf1**	ENSMUSG00000020053	Growth Factors & Signaling	429.5	-0.37	1.29	0.0366	0.0727	No	ns
**Igf2**	ENSMUSG00000048583	Growth Factors & Signaling	1521.8	-0.135	1.1	0.4258	0.5308	No	ns
**Hgf**		Growth Factors & Signaling						Not detected	
**Cdkn1a**	ENSMUSG00000023067	Cell Cycle Regulators	4241.5	2.661	6.32	4.35E-41	7.21E-38	**Yes**	↑
**Ccnd1**	ENSMUSG00000070348	Cell Cycle Regulators	156.7	-2.067	4.19	2.13E-10	4.91E-09	**Yes**	↓
**Ccnd2**	ENSMUSG00000000184	Cell Cycle Regulators	340.9	-1.132	2.19	3.32E-10	7.22E-09	**Yes**	↓
**Ccne2**	ENSMUSG00000028212	Cell Cycle Regulators	269.2	-0.774	1.71	4.00E-06	3.13E-05	**Yes**	↓
**Ccnd3**	ENSMUSG00000034165	Cell Cycle Regulators	500.5	-0.4	1.32	0.0017	0.0057	No	ns
**Cdkn1b**	ENSMUSG00000003031	Cell Cycle Regulators	1765.9	0.226	1.17	0.1288	0.2045	No	ns

**Cdkn1c**	ENSMUSG00000037664	Cell Cycle Regulators	1273.7	-0.082	1.06	0.6809	0.7577	No	ns
**Ccne1**		Cell Cycle Regulators						Not detected	
